# The LINC01119-SOCS5 axis as a critical theranostic in triple-negative breast cancer

**DOI:** 10.1038/s41523-021-00259-z

**Published:** 2021-05-31

**Authors:** Zhenbo Tu, Johannes Schmoellerl, Odette Mariani, Yurong Zheng, Yi Hu, Anne Vincent-Salomon, Antoine E. Karnoub

**Affiliations:** 1 Department of Pathology and Cancer Center, Beth Israel Deaconess Medical Center, Harvard Medical School, Boston, MA USA; 2 Institut Curie, Paris Cedex 05, France; 3 Harvard Stem Cell Institute, Cambridge, MA USA; 4 Broad Institute of MIT and Harvard, Cambridge, MA USA

**Keywords:** Cancer, Breast cancer

## Abstract

The development of triple-negative breast cancer (TNBC) is critically regulated by certain tumor-microenvironment-associated cells called mesenchymal stem/stromal cells (MSCs), which we and others have shown promote TNBC progression by activating pro-malignant signaling in neighboring cancer cells. Characterization of these cascades would better our understanding of TNBC biology and bring about therapeutics that eliminate the morbidity and mortality associated with advanced disease. Here, we focused on the emerging class of RNAs called long non-coding RNAs or lncRNAs and utilized a MSC-supported TNBC progression model to identify specific family members of functional relevance to TNBC pathogenesis. Indeed, although some have been described to play functional roles in TNBC, activities of lncRNAs as mediators of tumor-microenvironment-driven TNBC development remain to be fully explored. We report that MSCs stimulate robust expression of LINC01119 in TNBC cells, which in turn induces suppressor of cytokine signaling 5 (SOCS5), leading to accelerated cancer cell growth and tumorigenesis. We show that LINC01119 and SOCS5 exhibit tight correlation across multiple breast cancer gene sets and that they are highly enriched in TNBC patient cohorts. Importantly, we present evidence that the LINC01119-SOCS5 axis represents a powerful prognostic indicator of adverse outcomes in TNBC patients, and demonstrate that its repression severely impairs cancer cell growth. Altogether, our findings identify LINC01119 as a major driver of TNBC development and delineate critical non-coding RNA theranostics of potential translational utility in the management of advanced TNBC, a class of tumors in most need of effective and targeted therapy.

## Introduction

Breast cancer is one of the world’s leading diagnosed cancers with >2 million new cases identified across 20 geographical regions in 2018, including >300,000 in the US alone^1^. Although scientific and technological advances have allowed for better clinical management of the disease with increasingly ameliorated patient survival odds, breast-cancer-related mortality rates remain the second-highest of all cancers worldwide and are estimated at >40,000 US deaths in 2019^2^. These numbers highlight the pressing clinical need for continued research into breast cancer etiology, pathogenesis, and treatment.

Based on molecular and pathological determinations, breast cancer is divided into three main subclasses with differing clinical management and prognostic assessments: the estrogen receptor (ER) and progesterone receptor (PR)-positive tumors (called luminal subtypes), the human epidermal growth factor receptor 2 (HER2 or ERBB2) enriched tumors (called HER2), and the ER-negative, PR-negative, and HER2-negative tumors, also called triple-negative breast cancers (TNBCs)^3–6^. Among these three classes, patients identified with TNBC exhibit the worst overall outcome^7^. This is due to several parameters that include early onset of TNBC (which tends to occur in younger women), its preponderance in underprivileged ethnic/racial groups with limited access to healthcare, and an aggressive disease pathology with a predisposition for early metastasis^2,8,9^. In addition, TNBCs lack targeted therapies, and chemotherapeutic agents, such as taxanes or anthracyclines, which form the backbone of their clinical management^2^ are insufficient in controlling the disease in the majority of TNBC cases, especially when it spreads^10^. Increased molecular understanding of TNBC biology thus stands to provide new avenues for more effective and less toxic medicaments that can eliminate the morbidity and mortality associated with advanced and refractory patients.

TNBC development is tightly regulated by certain tumor-microenvironment-associated fibroblastoid cells called mesenchymal stem/stromal cells (MSCs) that others and we have shown play determining roles in exacerbating tumor malignancy^11–13^. While the mechanistic details of such influences have not been cataloged in their entirety, what is clear is that direct heterotypic interactions between MSCs and cancer cells represent the major driving force underlying MSC pro-malignant functions^14^. In these findings, physical MSC-cancer-cell contacts initiate complex networks of both coding and non-coding RNA mediators that work in singular and/or in tandem to trigger critical programs within cancer cells, such as epithelial-mesenchymal transition^15^ or cancer stem cell-ness^16^, leading to disease growth, spread, and therapy resistance^17^. These and many other similar findings^14^ emphasize the critical roles MSCs play in breast cancer in general and in TNBC in particular and highlight the utility of the MSC-supported tumor progression model in equally delineating disease regulators and vulnerabilities.

We most recently leveraged the MSC-TNBC-cell co-culture model to gain further mechanistic insights into the molecular mechanisms that promote TNBC development. Specifically, we focused on investigating the involvement of a particular family of regulatory non-coding RNAs—the long non-coding RNAs (lncRNAs)—as drivers of TNBC pathology. Indeed, lncRNAs represent a family of >200 nucleotide-long transcribed RNAs with described roles in primarily regulating gene expression in normal physiology and in disease settings, including cancer^18,19^. Whereas activities of individual lncRNAs of relevance to TNBC biology are being identified at a fast pace^20^, functions for the overwhelming members of the family are still outstanding. We found that MSCs induced robust LINC01119 expression in neighboring TNBC cells, which was sufficient, on its own, in promoting cancer cell growth in multiple contexts in vitro, as well as tumorigenesis in immune-compromised mice. At the molecular level, we demonstrated that LINC01119 was indeed a non-coding RNA and that it functioned via upregulating pro-tumorigenic activities of SOCS5, a member of the suppressor of cytokine signaling family of proteins. Importantly, LINC01119 and SOCS5 were critical for cellular growth, and we found that the LINC01119-SOCS5 axis associated tightly with clinical TNBC and that it served as a powerful prognosticator of poor patient outcome.

## Results

### LINC01119 is induced in MSC-stimulated TNBC cells and is tightly associated with clinical disease

**Fig. 1:**
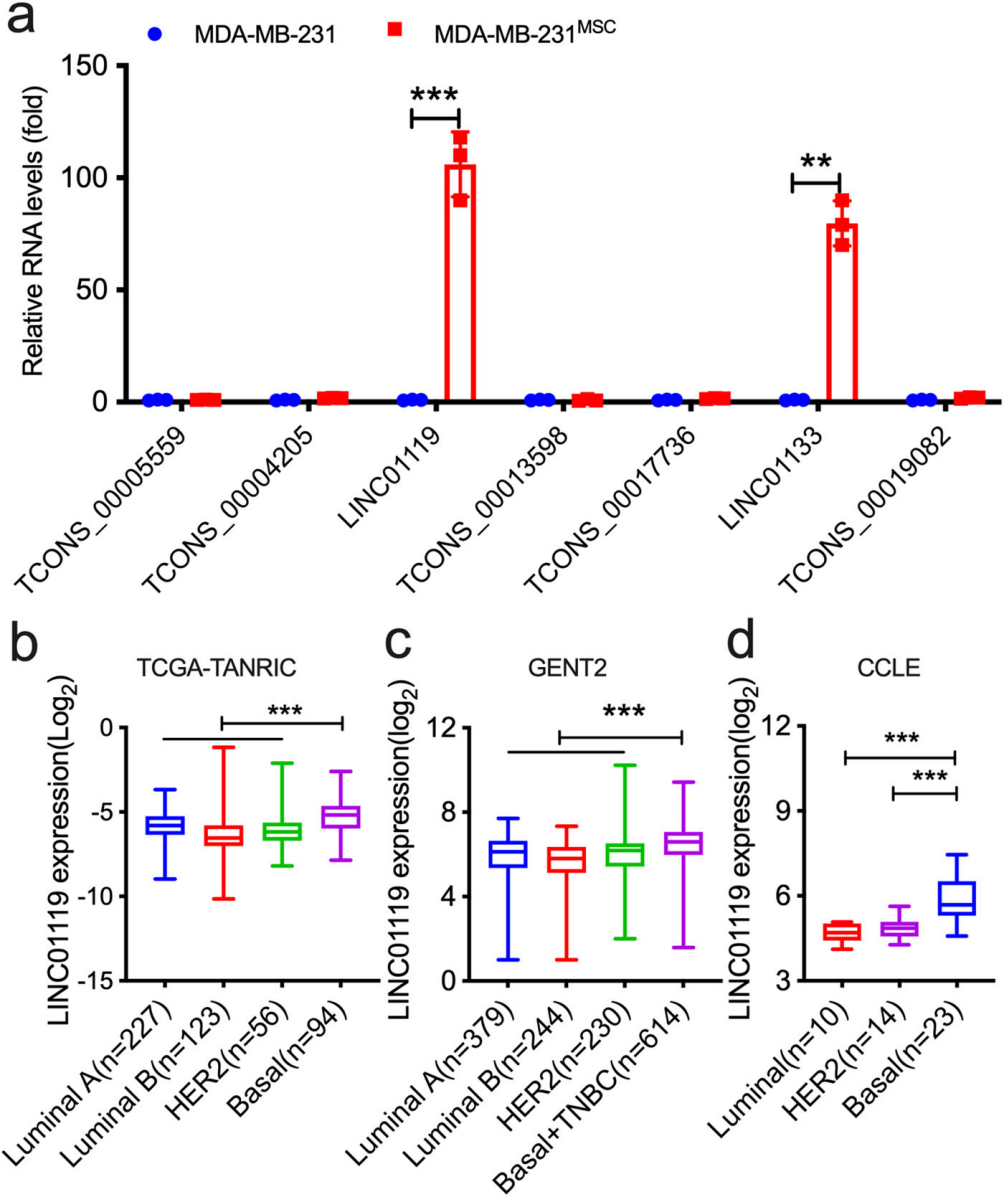
MSCs upregulate LINC01119 in admixed breast cancer cells. **a** qRTPCR measurements (meanâ€‰±â€‰SD of *n*â€‰=â€‰3) of indicated lncRNAs in sorted MDA-MB-231 cells recovered by FACS from 72-hr co-cultures with BM-MSCs (MDA-MB-231^MSC^). Sorted MDA-MB-231 cells cultured alone served as controls. **b**–**d** LINC01119 level (Log_2_) in TCGA data (from TANRIC database) analyzed by significance analysis of microarrays (SAMs) (**b**), in clinical breast cancer subtypes from GENT2 database (**c**), and in cancer cell lines representing different breast cancer subtypes in CCLE (**d**). Box-and-whisker plots represent the median (centerline) and inter-quartile range (IQR; box). The whiskers extend up to 1.5 times the IQR from the box to the smallest and largest points.

To identify lncRNAs of potential relevance to TNBC pathogenesis, we mined Affymetrix-based analyses in which we compared the gene expression profiles of GFP-labeled MDA-MB-231 TNBC cells recovered by FACS from 3-day co-cultures with human BM-MSCs versus that of sorted naïve GFP-BCC counterparts cultured alone as controls^15^ (Supplementary Fig. 1a). These efforts led to the identification of 7 lncRNAs that were upregulated >1.5 folds with a *q*-value threshold (false-discovery rate) of ~10% and with >98% probe specificity to the designated transcript (Supplementary Fig. 1b). qRTPCR-based validation of these candidates revealed mild inductions of certain of these lncRNAs in the MSC-stimulated cells, such as TCONS_00019082_1 or TCONS_00004205_1, and stronger inductions in others, such as LINC01133 (Fig. 1a), a lncRNA we previously characterized^21^ (Fig. 1a). Interestingly, however, the strongest induction was observed for LINC01119, which exhibited >100-fold upregulation in MSC-stimulated cells (Fig. 1a). As a novel lncRNA with previously undescribed functions in TNBC (or cancer in general), LINC01119 particularly attracted our attention.

LncRNAs show tissue and cell type-specific expression^18,19^. To explore if LINC01119 associated preferentially with clinical TNBC, we probed its expression levels in The Atlas of Non-Coding RNA In Cancer (TANRIC), a large RNA-seq database largely based on The Cancer Genome Atlas (TCGA) information. Here, we found that LINC01119 (as estimated by position chr 2: 47055003-47086145) was indeed significantly elevated in Basal clinical subsets when compared to luminal A/B or HER2 specimens (Fig. 1b). Similar patterns were observed when we examined the GENT2 database, which harbors >200 patient-derived specimens per breast cancer subtype (Fig. 1c). As the aforementioned RNA material was generated from specimens that undoubtedly contained not only carcinoma cells, but their stromal components as well, we were interested in determining if LINC01119 was enriched in cancer cells per se. For this purpose, we investigated LINC01119 expression in breast cancer cells profiled in the Cancer Cell Line Encyclopedia (CCLE, GSE36133; Supplementary Fig. 2a and Fig. 1d), and found it to be significantly associated with Basal A/B cells as well (a more definitive enrichment for LINC01119 within subsets of TNBC could not be demonstrated, although there was a tendency for its enrichment in M/MSL cells (Supplementary Fig. 2b). These results indicated that LINC01119 is enriched in TNBC experimental models and in clinical tumor tissues.

**Fig. 2:**
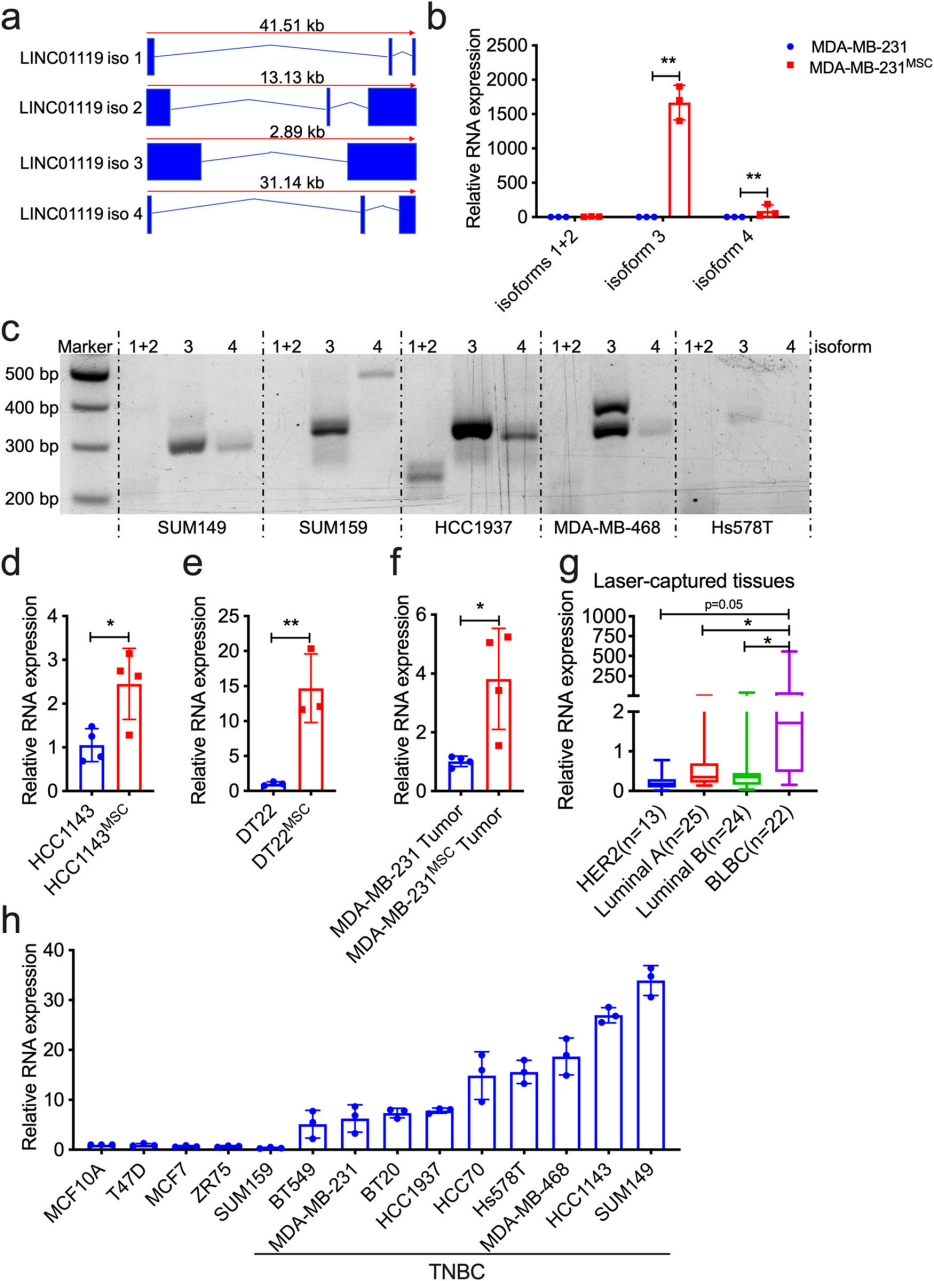
MSCs specifically upregulate LINC01119 isoform-3 in TNBC cells. **a** Schematic of LINC01119 isoforms derived from HUGO database. **b** qRTPCR measurements (mean ± SD of *n* = 3) of LINC01119 isoforms in MDA-MB-231^MSC^ versus controls. **c** Representative (*n* > 3) agarose-gel resolution of semi-quantitative PCR of LINC01119 isoforms in SUM149, SUM159, HCC1937, MDA-MB-468, and Hs578T cells. (**d**–**f**) qRTPCR measurements (mean ± SD of *n* > 3) of LINC01119 isoform-3 in HCC1143^MSC^ versus controls (**d**), in primary DT22^MSC^ versus controls (**e**), and in MDA-MB-231 cells recovered from human-MSC-containing tumor xenografts (**f**). **g** LINC01119 isoform-3 qRTPCR on dissected cancer cells lifted off slides of the indicated tumor samples. Box-and-whisker plots represent the median (centerline) and inter-quartile range (IQR; box). The whiskers extend up to 1.5 times the IQR from the box to the smallest and largest points. Normal breast tissue was used as a control. **h** LINC01119 expression (mean ± SD of 2^−∆∆CT^; compared with MCF10A; *n* = 3) in indicated breast cancer cells. LINC01119 levels were normalized to 18 S.

LINC01119 expression is considered to be of relatively low abundance, with the highest levels recorded in tissues such as the ovary or brain (Supplementary Fig. 3a–c). It emanates from chromosome 2p21 and its locus codes for 4 isoforms: TCONS_00003666 (isoform 1), TCONS_00003667 (isoform 2), TCONS_00002647 (isoform 3; the one annotated in the Affymetrix array) and TCONS_00002699 (isoform 4) (Fig. 2a). As lncRNA primary sequence dictates secondary structure, which in turn dictates function^22,23^, we sought to empirically identify the specific LINC01119 isoforms that were particularly induced in MSC-stimulated cells. Here, isoform-specific qRTPCR analyses using primers specific for isoform 1/2, isoform 3, and isoform 4 (Supplementary Fig. 4) revealed significant multifold induction of isoform 3 when compared to isoforms 1, 2, and 4 (Fig. 2b), which we determined was the most predominant endogenous isoform of LINC01119 across TNBC cells (Fig. 2c). Furthermore, BM-MSC co-culture with established TNBC cells, such as the cell line HCC1143, or with primary TNBC cells, such as DT22 cells^24^, equally led to significant 2-fold and 15-fold inductions of LINC01119 isoform 3 in the admixed cancer cells, respectively (Fig. 2d, e), suggesting that LINC01119 induction by BM-MSCs was not particular to MDA-MB-231 cells. In addition, we found that cancer cells recovered from human BM-MSC-containing Nude-mouse-derived xenografts^16^ also exhibited 3-4-fold stimulation of LINC01119 isoform 3 expressions compared to controls (Fig. 2f), indicating that this induction also occurred in vivo. Finally, since the Affymetrix probe set used to identify LINC01119 expression (probe 230799_at) in clinical specimens cannot distinguish between the different LINC01119 isoforms, we conducted laser-capture dissection of breast cancer cells from breast cancer samples of basal-like breast cancers (BLBC), luminal A, luminal B, and HER2 tumors and processed their RNA for qRTPCR using primers specific for isoform 3. Here too, we found several-fold enrichment of LINC01119 in carcinoma cells derived from BLBCs versus other types (Fig. 2g), consistent with our earlier observations using TANRIC (Fig. 1b) and with specific qRTPCR on a series of TNBC and non-TNBC cell lines (Fig. 2h). Together, these results prompted us to investigate the functional contributions of LINC01119 isoform 3 (heretofore LINC01119) to TNBC development.

### LINC01119 promotes pro-tumorigenic traits in TNBC cells

**Fig. 3:**
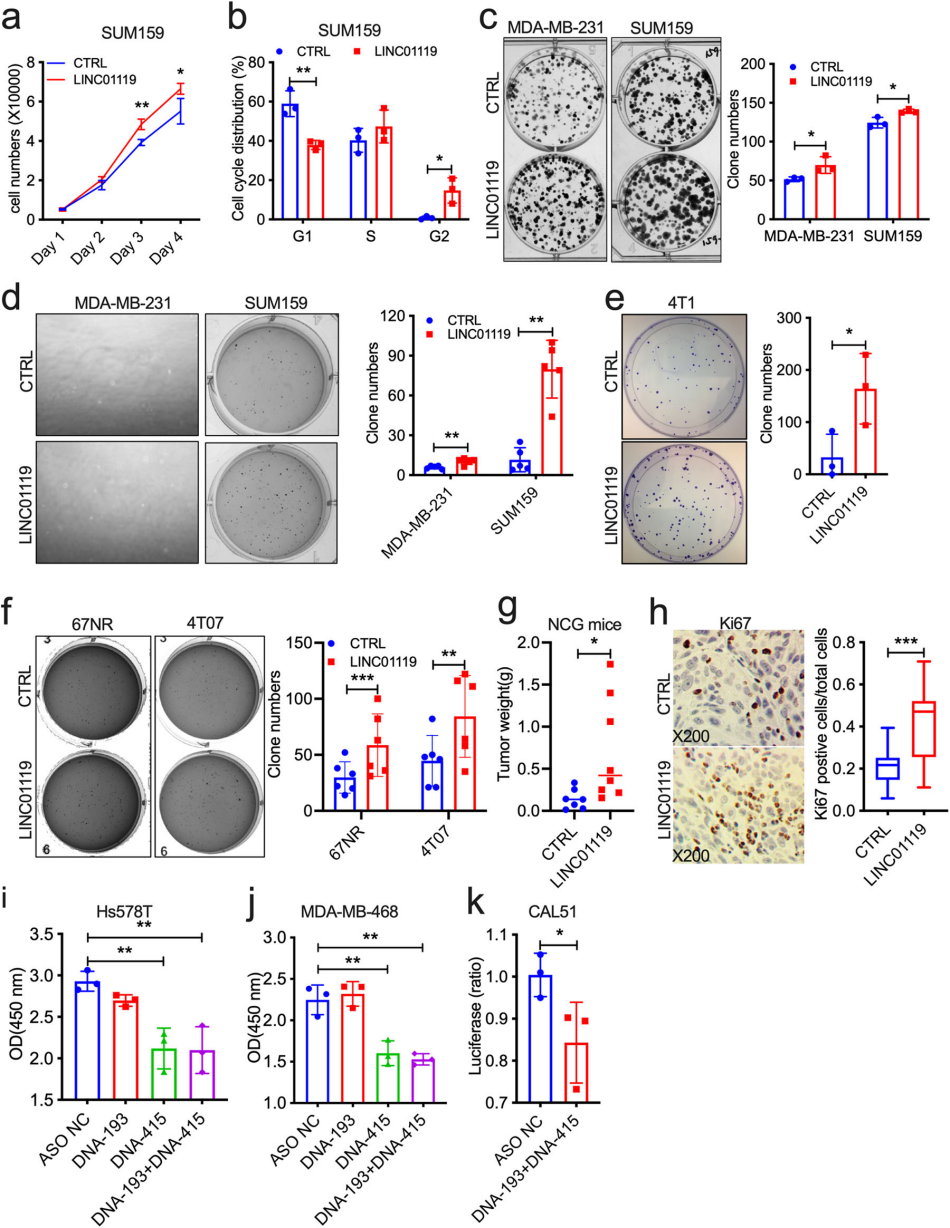
LINC01119 promotes oncogenic functions. **a**, **b** Proliferation (**a**) and cell cycle (**b**) of LINC01119-overexpressing SUM159 cells compared to controls at the indicated days (mean ± SD of *n* = 3). **c** Left: representative images of colony-formation assays on the indicated MDA-MB-231 and SUM159 groups. Right: ImageJ quantitation of colony numbers in Left displayed as mean ± SD of *n* = 3. **d** Left: representative images of 3D anchorage-independent growth of the indicated MDA-MB-231 and SUM159 cell lines. Right: ImageJ quantitation of colony numbers in Left displayed as mean ± SD of *n* > 3. **e** Left: representative images of colony-formation assays on the indicated mouse 4T1 groups. Right: ImageJ quantitation of colony numbers in Left displayed as mean ± SD of *n* = 3. **f** Left: representative images of 3D anchorage-independent growth of the indicated mouse 67NR and 4T07 cell lines. Right: ImageJ quantitation of colony numbers in Left displayed as mean ± SD of *n* > 3. **g** Weight of the indicated SUM159 tumors grown orthotopically in NCG mice after 42 days (*n* > 5 per group). **h** Left: representative images of immunohistochemistry of Ki67 in tumor tissues in g. Right: quantitation of Ki67 positive cells in Left displayed as box-and-whisker plots representing the median (centerline) and inter-quartile range (IQR; box). The whiskers extend up to 1.5 times the IQR from the box to the smallest and largest points. **i**, **j** Proliferation (mean ± SD of *n* = 3) of Hs578T (**i**) and MDA-MB-468 (**j**) cells transfected with controls (NC) or with LINC01119 ASOs measured using WST-1 assay after 48 h. **k** Proliferation (mean ± SD of *n* = 3) of CAL51 cells transfected with control or with a combination of LINC01119 ASOs measured using CellTiter after 48 h.

To probe the functions of LINC01119 in TNBC biology, we cloned LINC01119 NR_024452 (which corresponds to isoform 3) and tested the effects of its overexpression in multiple TNBC cell lines (Supplementary Fig. 5a). While LINC01119 did not enhance certain malignant traits of human cancer cells, such as resistance to suspension-induced cell death (anoikis; Supplementary Fig. 5b), it did promote significant increases in cell proliferation (Fig. 3a) and promoted cell cycle progression in SUM159 cells (Fig. 3b), enhanced the clonogenic growth of both SUM159 and MDA-MB-231 cells in 2D (Fig. 3c), as well as triggered 2-9-fold increase in the anchorage-independent growth of these cells in soft-agar assays (Fig. 3d). LINC01119 promoted similar growth phenotypes in mouse carcinoma cells too. Indeed, it caused a ~4-fold increase in 4T1 clonogenic growth in vitro (Fig. 3e), and about doubled the ability of 67NR and 4T07 murine mammary TNBC cells to grow in anchorage-independence (Fig. 3f). Most importantly, LINC01119-overexpressing SUM159 cells formed larger orthotopic tumors in immunocompromised NCG mice (Fig. 3g), which exhibited a ~2-fold increase in their Ki67-positivity compared to control tumors (Fig. 3h). Interestingly, antisense oligonucleotides (ASO)-mediated inhibition of endogenous LINC01119 (Supplementary Fig. 6a) led to significant reductions in cellular growth in Hs578T, in MDA-MB-468, and in CAL51 TNBC cells too (Fig. 3i–k), altogether underscoring the critical pro-oncogenic abilities of LINC01119 both in vitro and in vivo.

### LINC01119 is a non-coding RNA with predominant cytoplasmic localization

**Fig. 4:**
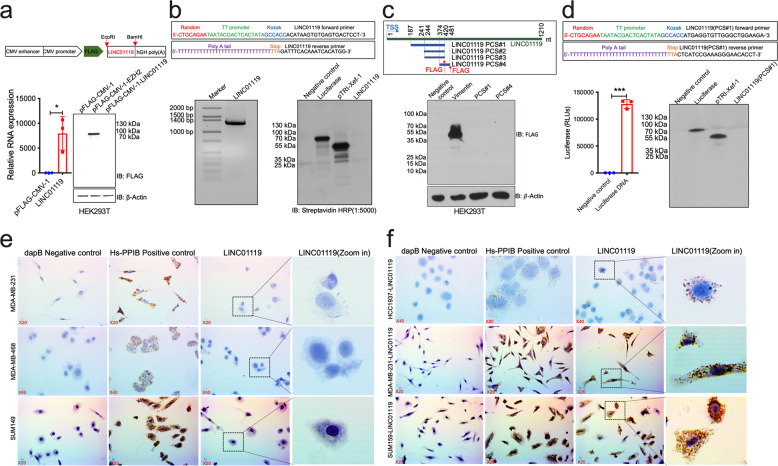
LINC01119 is a bona fide non-coding RNA located in the cellular cytoplasm. **a** Upper: schema of LINC01119 plasmid template for *in-cell* transcription/translation. Bottom: qRTPCR (mean ± SD of *n* = 3) (left) and Western blot (right) of RNA and lysates derived from HEK293T cells transiently transfected with LINC01119 or indicated controls. **b** Upper: schema of 5’ and 3’ LINC01119 primers utilized in in vitro transcription reactions. Bottom Left: in vitro transcribed LINC01119. Bottom Right: SDS-PAGE on in vitro translated luciferase, pTRI-Xef-1, and LINC01119. **c** Upper: schema of LINC01119 putative coding sequences. Bottom: Western blot analysis of lysates of HEK293T cells transiently transfected with empty pFLAG-CMV-1, pFLAG-CMV-1-Vimentin, PCS#1 and #4. **d** Upper: schema of 5’ and 3’ LINC01119 PCS#1 with primers utilized in in vitro transcription reactions. Bottom Left: Luciferase activity (mean ± SD of *n* = 3) of the successfully transcribed and translated luciferase used as a positive control for transcription. Bottom Right: SDS-PAGE on in vitro translated luciferase, pTRI-Xef-1, and LINC01119 PCS#1. **e**, **f** Localization of LINC01119 in control MDA-MB-231, MDA-MB-468, and SUM149 cells (**e**) and in HCC1937, MDA-MB-231 and SUM159 cells stably over-expressing LINC01119 (**f**) determined by RNAscope; dapB and Hs-PPIB served as negative and positive controls, respectively. Images shown are representatives of *n* > 3.

We proceeded to identify how LINC01119 exerted its functional activities in support of tumor cell growth, focusing first on determining its protein-coding potential. Indeed, previous studies indicated that certain lncRNAs, such as LINC00961^25^ or HOXB-AS3^26^, in fact, coded for small peptides with proven functional activities. We, therefore, set out to determine if LINC01119 open reading frame (ORF) in effect produced a protein product, and did so using several approaches. First, we inserted a FLAG-tag at the 5-prime end of the LINC01119 sequence and expressed the construct by transfection into HEK293T cells; however, immunoblotting of cell lysates with FLAG antibodies detected no product for LINC01119 in these cells, in contrast to FLAG-tagged EZH2, which was used as a protein-coding positive control (Fig. 4a). Second, we followed up on these *in-cell* results by conducting further experiments assessing LINC01119 protein-coding potential in stringently controlled experiments in vitro. Here, we inserted T7 promoter and Kozak sequences at the 5-prime end of LINC01119 (Fig. 4b) and conducted in vitro transcription followed by in vitro translation analyses with Biotin-labeled amino acids. While LINC01119 sequence was adequately transcribed, streptavidin-based Western immunoblotting could not detect a protein product for LINC01119 when compared to translated Luciferase or Xef1 proteins, detected at 61 and 50 kDa, respectively (Fig. 4b). Third, having found LINC01119 to be amongst a large set of identified ribosome-associated lncRNAs^27^, and that it was predicted, albeit with very low probability, to code for small peptides derived from 4 putative coding segments (PCS; Fig. 4c), we proceeded to test this notion further. Since PCS-1, PCS-2, and PCS-3 share an identical 3-prime sequence, we cloned both PCS-1 and PCS-4 sequences in-frame into pFLAG-CMV-1 plasmid and expressed them in HEK293T cells. Here, no FLAG-tagged protein product was detected in the corresponding cell lysates (Fig. 4c). In addition, we specifically cloned PCS-1 downstream of the T7 promoter and Kozak and checked its protein-coding potential in vitro (Fig. 4d). However, no protein product was detected subsequent to in vitro transcription and translation of PCS-1 (Fig. 4d), further confirming the non-coding nature of LINC01119. Finally, since lncRNA functions are linked to their cellular localization^28,29^, we determined LINC01119 cellular distribution patterns via in situ hybridization with LINC01119-specific probes using RNAScope^30,31^. These results showed a predominant cytoplasmic localization for both endogenous (in SUM149 cells, one of the most enriched for LINC01119 (Fig. 2h)), and exogenous LINC01119 expressed in HCC1937, SUM159, and MDA-MB-231 cells (Fig. 4e, f). Collectively, these results demonstrated empirically that LINC01119 is a non-coding RNA that functions predominantly from the cytoplasm.

### LINC01119 stimulates SOCS5 in TNBC cells

**Fig. 5:**
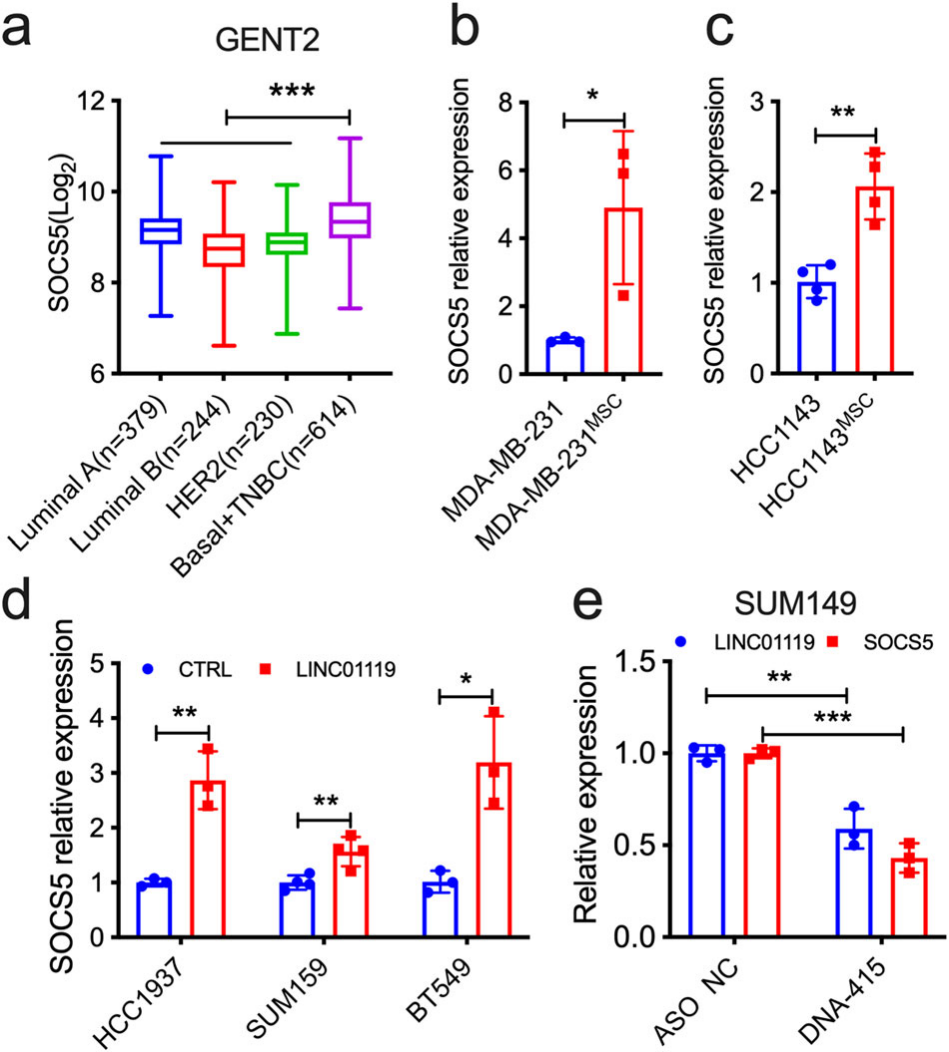
LINC01119 regulates SOCS5. **a** SOCS5 levels in clinical breast cancer from GENT2 database analyzed by SAMs. Box-and-whisker plots represent the median (centerline) and inter-quartile range (IQR; box). The whiskers extend up to 1.5 times the IQR from the box to the smallest and largest points. **b**, **c** qRTPCR measurements of SOCS5 levels in the indicated MDA-MB-231^MSC^ (**b**) and HCC1143^MSC^ (**c**) cells. **d** qRTPCR measurements of SOCS5 in the indicated HCC1937, SUM159, and BT549 cells stably over-expressing LINC01119. **e** qRTPCR measurements of LINC01119 and SOCS5 in SUM149 cells 48 h after transfection with control or anti-LINC01119 ASOs. For analyses in **b**–**e**, mean ± SD of *n* = 3 are shown.

To determine more specifically how LINC01119 exerted its pro-tumorigenic activities in TNBC, we conducted large-scale gene expression analyses looking for genes whose expression most closely correlated with that of LINC01119 in the breast cancer cohorts found in TANRIC (Supplementary Fig. 7a). When the top 5 genes from this list were cross-compared to LINC01119 co-correlated genes across 23 TNBC cell lines profiled in CCLE, only one gene—suppressor of cytokine signaling 5 or SOCS5—positively correlated with LINC01119 at *R* > 0.45 and *p* < 0.05 across these 2 databases (Supplementary Fig. 7b). Indeed, SOCS5 associated preferentially with Basal and TNBC clinical specimens when compared to Luminal A, Luminal B, and HER2 subtypes from the GENT2 database (Fig. 5a), subsets in which LINC01119 was also more preponderant (Fig. 1), and we observed similar connections between LINC01119 and SOCS5 using our experimental systems. First, we found that MSCs, which stimulated LINC01119 in admixed cancer cells (Figs. 1 and 2), also caused ~5 and ~2-fold enrichment of SOCS5 expression in co-cultured MDA-MB-231 (Fig. 5b) and HCC1143 cells (Fig. 5c), respectively. In addition, LINC01119 was sufficient, on its own, in causing 2-3-fold induction of SOCS5 expression in HCC1937, SUM159, BT549, and MDA-MB-231 TNBC cells (Figs. 5d and S8). Conversely, 50% downregulation of LINC01119 levels in the LINC01119-high SUM149 cells using ASOs (Supplementary Fig. 6a) caused >50% reduction in the endogenous basal SOCS5 levels (Fig. 5e). It is worthy to add that we probed whether LINC01119 induction of SOCS5 involved transcriptional or posttranscriptional mechanisms. Here, we found that the rates of SOCS5 mRNA or protein decay in actinomycin D or cycloheximide-treated cells were identical between control and LINC01119-overexpressing cells (Supplementary Fig. 7c, d), indicating no discernable effect of LINC01119 on either mRNA or protein stability of SOCS5. These results collectively suggested that LINC01119 is both sufficient and necessary as an upstream regulator of *de novo* SOCS5 transcription.

SOCS5 belongs to the family of suppressors of cytokine signaling proteins, which are broadly categorized as negative regulators of the Janus Activated Kinase (JAK) and Signal Transducer and Activator of Transcription (STAT) pathway and are thought to exert major roles in attenuating receptor-initiated signaling^32–34^. In particular, SOCS5 has been shown to inhibit JAK1/2 and STAT1/3 phosphorylation in different cellular contexts^35–38^, so we probed the phosphorylation status of these proteins in SUM159 and MDA-MB-231 cells stably expressing LINC01119 in order to determine if SOCS5 downstream activities were also revved up in accompaniment to SOCS5 mRNA and protein induction; however, we did not find any inhibition of phospho-JAK1, phospho-JAK2, phospho-STAT1, or phospho-STAT3 by LINC01119 (Supplementary Fig. 8a–d). We therefore expanded our investigation to include all other members of the JAK and STAT proteins. We observed unique and reproducible downregulation of phospho-STAT6 in both cell lines under serum-starved and non-serum-starved conditions (Supplementary Fig. 8a–d). These results were confirmed in cancer (and non-cancer) cells transiently overexpressing exogenous human SOCS5 (Supplementary Fig. 8e), echoing prior reports in which SOCS5 was described to inhibit STAT6 activation in dendritic cells^39^ or in T helper (Th) subsets^40^. Notably, and in genetic support of these observations, we found that STAT6 was most depleted in TNBC specimens in TCGA (Supplementary Fig. 8f), as well as in TNBC-rich p53 mutant samples (Supplementary Fig. 8g) and that SOCS5 and STAT6 exhibited negative correlation in BLBC and ER-negative clinical specimens (Supplementary Fig. 8h, i). Together, these observations indicated that LINC01119-induced SOCS5 is active in TNBC cells.

### SOCS5 is a critical regulator of TNBC cell growth

**Fig. 6:**
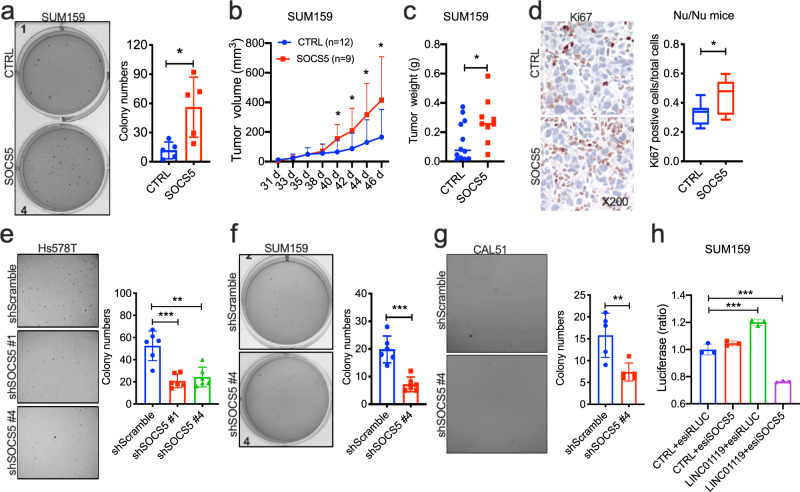
Critical role for SOCS5 in TNBC cell growth. **a** Left: representative images of anchorage-independent growth patterns of controls versus SUM159 cells stably expressing human SOCS5. Right: ImageJ quantitation of colony numbers in Left displayed as mean ± SD of *n* = 5. **b** Growth kinetics of indicated orthotopic tumors derived from SUM159 controls (*n* = 12) or SUM159 cells expressing pLVX-SOCS5 (*n* = 9) in Nu/Nu nude mice measured by digital calipers with mean ± SD indicated per time point. **c** Weight of the indicated SUM159 tumors in B with the median shown. **d** Left: representative images of immunohistochemistry of Ki67 in tumor tissues in **c**; Right: quantitation of Ki67 positive cells in Left displayed as box-and-whisker plots representing the median (centerline) and inter-quartile range (IQR; box). The whiskers extend up to 1.5 times the IQR from the box to the smallest and largest points. **e** Left: representative images of anchorage-independent growth patterns of Hs578T cells stably expressing sh scramble control, shSOCS5#1 and shSOCS5#4. Right: ImageJ quantitation of colony numbers in Left displayed as mean ± SD of *n* > 3. **f** Left: representative images of anchorage-independent growth patterns of controls versus SUM159 cells stably expressing SOCS5 shRNA#4. Right: ImageJ quantitation of colony numbers in Left displayed as mean ± SD of *n* > 3. **g** Left: representative images of anchorage-independent growth patterns of controls versus SOCS5 shRNA#4 in CAL51 cells. Right: ImageJ quantitation of colony numbers in Left displayed as mean ± SD of *n* > 3. **h** Proliferation (mean ± SD of *n* = 3) of control or LINC01119-over-expressing SUM159 cells transfected with esiRLUC or esiSOCS5 measured using CellTiter after 72 h.

We proceeded to determine the functional roles for SOCS5 in TNBC cell growth. We found that overexpression of human SOCS5 in SUM159 (Supplementary Fig. 8 and Supplementary Fig. 9a) induced a ~5-fold increase in anchorage-independent growth in soft-agar (Fig. 6a), and generated tumors that grew significantly faster and larger in Nude mice (Fig. 6b, c), and with ~2-fold as many Ki67-positive carcinoma cells (Fig. 6d), indicating that SOCS5, similar to LINC01119, was sufficient, on its own, in promoting malignant growth both in vitro and in vivo. To test the essentiality of SOCS5, we screened shRNAs against SOCS5 and selected two hairpins that resulted in ~70% inhibition of its mRNA (Supplementary Fig. 9b). Expression of these two shRNAs in Hs578T TNBC cells resulted in >50% inhibition of the cells ability to grow in soft-agar conditions (Supplementary Figs. 9c and 6e), suggesting that SOCS5 performed essential pro-tumorigenic functions in these contexts. Identical results were obtained in two additional TNBC models, SUM159 and CAL51, in which shSOCS5#4 caused ~50% inhibition of growth in soft-agar (Supplementary Figs. 9d, e, and Figs. 6f, g). Most importantly, knockdown of SOCS5 by enhanced siRNA (esiRNA; Supplementary Fig. 9f) abrogated LINC01119-induced cell proliferation (Fig. 6h), in support of the notion that SOCS5 is an essential partner of LINC01119 that performs critical functions in TNBC cell growth.

### The LINC01119-SOCS5 axis is prognostic of poor patient outcome

**Fig. 7:**
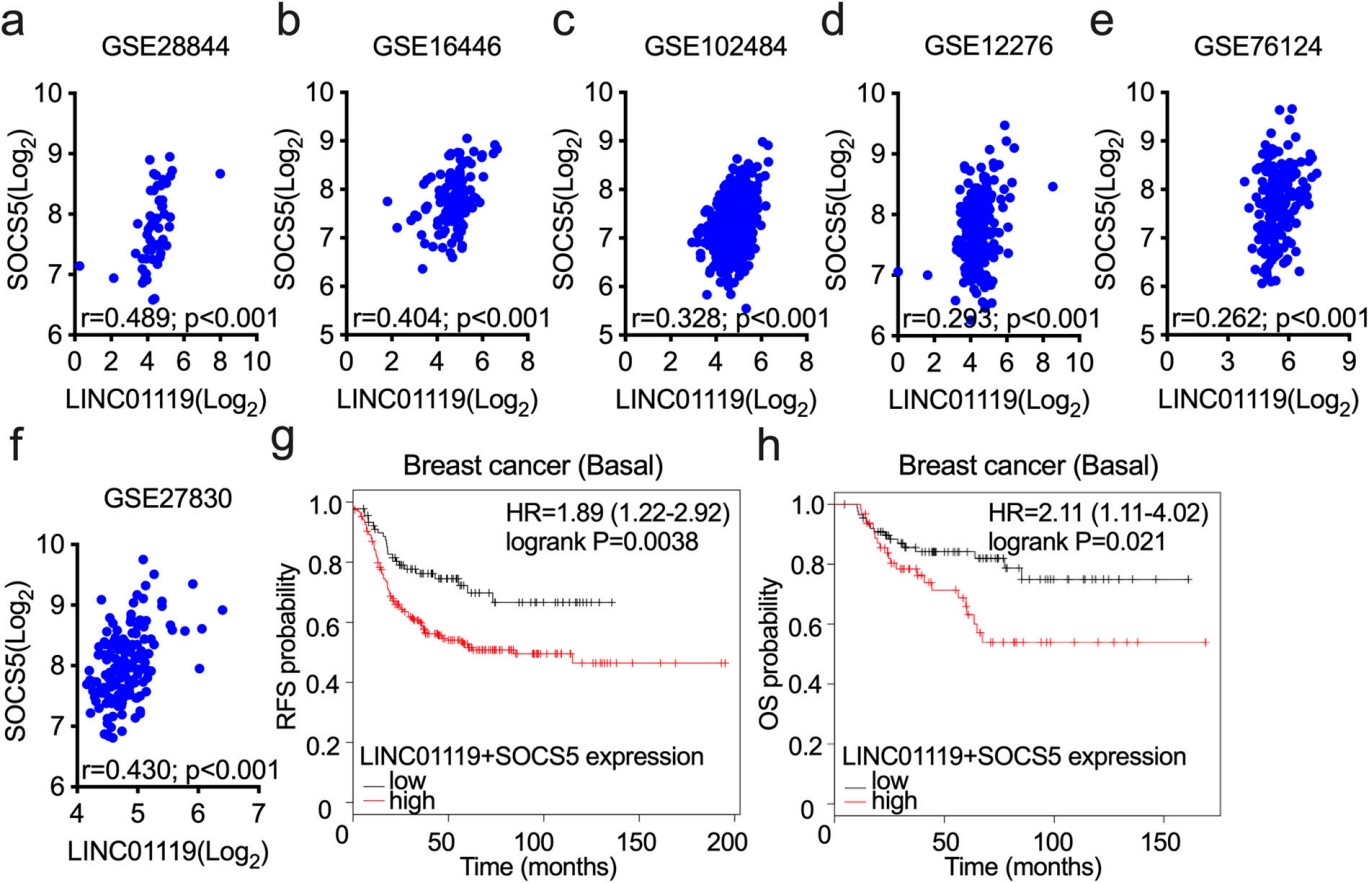
LINC01119-SOCS5 pathway is prognostic of poor patient survival. **a**–**f** Correlation of LINC01119 and SOCS5 in GSE28844 (**a**), GSE16446 (**b**), GSE102484 (**c**), GSE12276 (**d**), GSE76124 (**e**), and GSE27830 (**f**). **g** Kaplan-Meier analysis (kmplot.com) of relapse-free survival (RFS) based on the mean value of LINC01119 and SOCS5 in basal breast cancer (*n* = 544), the cutoff value of 301.5, probe 230799_at for LINC01119 and 209648_x_at for SOCS5. **h** Kaplan-Meier analysis of overall survival (OS) based on the mean value of LINC01119 and SOCS5 in basal breast cancer (*n* = 248), the cutoff value of 438, probe 230799_at for LINC01119 and 209648_x_at for SOCS5.

We next probed the clinical relevance of our findings across publicly accessible gene expression databases of breast cancer. We found that SOCS5 positively correlated with LINC01119 across multiple different clinical breast cancer datasets, which included GSE28844 (Fig. 7a), GSE16446 (Fig. 7b), GSE102484 (Fig. 7c), and GSE12276 (Fig. 7d). Similar findings were observed using the large TNBC cohort of Brown and colleagues (GSE76124)^41^; Fig. 7e) and in the BRCA1-mutant specimens of GSE27830 (Fig. 7f), tumors that have a high propensity to classify with triple-negative BLBCs. Furthermore, high expression levels of LINC01119 and SOCS5 associated with shorter relapse-free survival (RFS) in breast cancer patients in general (Fig. 7g) and poorer overall survival (OS) in those diagnosed with BLBC in particular (Fig. 7h). Altogether, these findings are consistent with a model in which the stroma-regulated LINC01119-SOCS5 axis assumes critical pro-malignant functions in TNBC development, and that it represents both a therapeutic target (Figs. 3 and 6) and a prognosticator of adverse patient outcome in disease management.

## Discussion

In the present work, we utilized the MSC:BCC co-culture model to reveal functions for lncRNAs in regulating TNBC development. Thus, we found that MSC-derived triggers induced LINC01119 expression in neighboring TNBC cells, which in turn stimulated SOCS5, leading to accelerated in vitro cancer cell growth, both in adhesion and in suspension, as well as accentuated in vivo tumor formation in xenografted mice. In addition, LINC01119 and SOCS5 expression were significantly enriched in several TNBC patient cohorts and exhibited substantial and tight correlation with one another across multiple breast cancer gene sets. Importantly, LINC01119/SOCS5 repression severely impaired cancer cell growth, and the pathway served as a powerful prognostic indicator of adverse outcomes in TNBC patients. Collectively, our findings have delineated previously undescribed non-coding theranostic elements of potential translational utility in TNBC management.

To our knowledge, this is the first report that describes cellular activities of LINC01119 and that begins to elucidate its functional downstream effectors. Although there are no previous links that have tied LINC01119 to malignancy, it is important to point out that LINC01119 is located on chromosome 2p, an arm noted by several groups to undergo aberrations in breast cancer^42,43^ and to exhibit elevated probability for harboring susceptibility loci for breast^44^ and other neoplasms, such as endometrial^45^ or renal^46^ carcinomas. In addition, several genes that map to the vicinity of LINC01119 on 2p21 have been implicated in advancing tumorigenesis and metastasis in multiple malignancies, such as PKCε^47^ or SOS1^48^. These observations, together with reports that underscore the ability of lncRNAs to regulate neighboring genes *in cis*^49^ suggest a potential role for LINC01119 in the etiology and development of cancers beyond TNBC. Notable amplifications of LINC01119 in a number of cancers across the TCGA, such as pancreatic, uterine, and lung cancers (Supplementary Fig. 10) would indeed be consistent with this hypothesis.

Prior work indicated that SOCS5 falls under the tight regulatory control of complex non-coding RNA networks that involve an expanding list of both microRNAs and lncRNAs. Indeed, several miRs have been described to directly suppress SOCS5 expression in a variety of cancer cells, such as miR-9 in prostate cancer^50^, miR-885 in colorectal cancer^51^, miR-301a in pancreatic cancer cells^33^, or miR-18, miR-25, and miR-589 in liver cancer^52,53^. In contrast, a handful of lncRNAs have been shown to induce SOCS5 expression in target cancer cells, and these include HAND2-AS1 in liver cancer^54,55^, FER1L4 in osteosarcoma^56^, TUSC7 in endometrial carcinoma^57^, MEG3 in oral squamous cell carcinoma^58^, and LINC00668 in glioma^59^. Interestingly, a unifying mechanism-of-action of such lncRNAs in SOCS5 regulation appears to involve lncRNA sponging SOCS5-specific microRNAs, such as miR-18a, miR-616, or miR-584d, thereby relieving otherwise suppressed SOCS5 levels. Similar high-stringency miRBase-based approaches we conducted using our own analyses, however, only identified miR-3689d as a potential SOCS5-miR that can be sponged by LINC01119, but our preliminary findings indicated that miR-3689d was not induced in our MSC-stimulated cancer cells^16^ and that it was not modulated in LINC01119 overexpressing cells at all (data not shown). These findings, in addition to observations that SOCS5 induction in LINC01119-over-expressing cells was not due to upregulations in its mRNA or protein stabilities (Fig. S7c–d), suggested that LINC01119-stimulated increases in SOCS5 likely involve *de novo* transcriptional stimulation. Here, we posit that cytoplasmic LINC01119 (Fig. 4) sequesters a transcriptional regulator that otherwise suppresses SOCS5 transcription. Verification of this hypothesis awaits the detailed elucidation of the molecular interactions of LINC01119.

SOCS5 functioned as a partner of LINC01119, and we found that it was both sufficient and necessary to promote TNBC cell growth across several TNBC cells and that it promoted tumor growth in nude mice. Although a role for SOCS5 as a promoter of TNBC pathogenesis has not been previously described per se, our results were surprising considering several reports describing SOCS5 as a suppressor of cancer traits in the context of malignancies that included T cell lineage acute lymphoblastic leukemia (T-ALL)^34^, pancreatic cancer^60^, hepatocellular carcinoma (HCC)^52^, or prostate cancer^50^. How SOCS5 mechanistically exerted these suppressive activities has largely been attributed to its ability to mediate negative feedback loops that downregulated membrane tyrosine kinases, especially EGFR^61,62^. In these models, SOCS5 is thought to associate with autophosphorylated EGFR to then assemble a complex containing elongin B/C and E3 ubiquitin ligase that then targets EGFR for proteasomal degradation^63,64^. Although SOCS5 is postulated to act on other targets, such as Shc-1 or YAP1, in similar manners^36,65^, there is no evidence that it acts on other growth factor receptors, such as FGF or NGF. Whether SOCS5 engages in similar interactions in our systems is presently unknown.

In spite of these reports, however, the pro-oncogenic roles we describe for SOCS5 in TNBC cells are in agreement with prior (albeit fewer) reports indicating tumor-promoting activities for SOCS5. These include findings in which SOCS5 levels were shown to be induced in cancerous versus normal breast cells (including the TNBC cell line HCC1937 we used here)^66^, and in which SOCS5 proved to be essential to the viability of other breast cancer cells, such as the HER2 cell line SKBR3^67^. Pro-malignant roles for SOCS5 were also observed in hepatocellular carcinoma as well, where SOCS5 inhibition induced autophagy and compromised lung metastasis of HCC cells, and where high SOCS5 levels were prognostic of poor patient outcome^68^. Why SOCS5 seemingly performs diametrically opposing functions in tumor pathogenesis across different cancer types, however, remains an outstanding question. Here, and in keeping with reported functions for SOCS proteins as suppressors of JAK/STAT pathways, we did find that SOCS5 (and LINC01119) inhibited the phosphoactivation of STAT6, but not the traditional targets STAT1 or STAT3. Of pertinence, STAT6 has been described to act as a tumor suppressor in breast cancer^69,70^, providing a possible mechanism-of-action of LINC01119/SOCS5 in driving TNBC cell growth by curtailing STAT6 activation. In support of this notion are genetic data demonstrating STAT6 negative correlation with both LINC01119 and SOCS5 in patient cohorts, and the fact that STAT6 downregulation was itself associated with TNBC and forecasted poor TNBC patient prognosis (Supplementary Fig. 8f–g, j). Hence, we posit that LINC01119 induces SOCS5 expression, which then inhibits STAT6, relieving its tumor-suppressive functions in TNBC cells. The extent to which this pathway operates identically in additional breast cancer subtypes (or other cancers altogether) will necessitate the detailed determination of cellular SOCS5 targets, partners, and mechanism-of-action in a context-dependent fashion.

A final note is that the nomenclature of lncRNAs has followed the norm of naming lncRNAs according to the closest gene in their locus, and it is for this reason that LINC01119 is also known as lncSOCS5. For LINC01119, the nomenclature also carries functional connotation since we have shown that LINC01119 regulated SOCS5 expression and that SOCS5 served critical roles in LINC01119-regulated activities. The fact that SOCS5 and LINC01119 are also syntenic in humans suggests that they may have genetic interactions beyond the functional ones we describe here and that LINC01119 and SOCS5 act as an axis (or a duo of sorts) in regulating downstream functions.

## Methods

### Cell cultures

Breast cancer cell lines MDA-MB-231, MDA-MB-468, HCC1937, BT20, HCC1143, BT549, and Hs578T cells were procured from American Type Culture Collection (ATCC). HCC70, T47D, ZR75, SUM149, CAL51, and SUM159 were obtained from A. Toker (Beth Israel Deaconess Medical Center, Boston, MA), 4T1, 67NR, 4T07, MCF7, and HEK293T cells from R. Weinberg (Whitehead Institute, Cambridge, MA), and MCF-10A from J. Brugge (Harvard Medical School, Boston, MA). Human primary breast cancer cells DT22 were a gift from D. El-Ashry (Sylvester Comprehensive Cancer Center, University of Miami Miller School of Medicine, Miami, FL)^24^. Bone marrow-derived human mesenchymal stem cells (BM-MSCs) were purchased from the Institute for Regenerative Medicine at Scott and White, Texas A&M Health Science Center (Temple, TX). Established cancer cell lines were cultured according to ATCC recommendations. DT22 cells and BM-MSCs were cultured in Dulbecco’s modified Eagle’s medium (DMEM) with 10%fetal bovine serum (FBS). MCF10A cells were cultured in DMEM/Ham’s F12 medium supplemented with 5% horse serum (Gibco BRL, Waltham, MA), insulin (10 μg/ml; Wisent Bioproducts, Saint-Jean Baptiste, QC), hydrocortisone (500 ng/ml; Sigma–Aldrich, St. Louis, MO), epidermal growth factor (EGF) (20 ng/ml; R&D, Minneapolis, MN), and cholera toxin (100 ng/ml; List Biological Laboratories, Campbell, CA).

### Gene expression analyses

Comparative gene expression profiling of MSC-stimulated cancer cells versus controls was performed as previously described ^21^. Briefly, BM-MSCs were mixed with GFP-labeled BCCs (at 3:1 ratio of MSC:cancer cell) and cultured in DMEM-10% FBS for 72 h with GFP-BCCs cultured alone serving as controls, as we previously described^15^. Cultures were subsequently washed with PBS, trypsinized, centrifuged for 3 min at 1200 rpm, and resuspended in ice-cold PBS. Suspensions were then passed through 70 µm filters and sorted for GFP-positivity using FACS Aria II (BD) with similarly processed control GFP-BCCs cultured alone used to determine sorting gates based on cell size and GFP fluorescence intensity. Gates were set to exclude cell debris, potential aggregates, and to collect cancer cells with the strongest GFP expression to ensure avoidance of GFP-negative MSC contamination. For Affymetrix analyses, 1 µg total RNA was recovered from FACS-isolated MDA-MB-231 cells, processed through library preparation using GeneChip HT One-Cycle cDNA synthesis kit and Genechip HT IVT Labeling kit (900687 and 900688; Affymetrix, Santa Clara, CA), and hybridized to HT-HG U133 2.0 Plus chip (900751; Affymetrix, Santa Clara, CA).

Gene-specific expression analysis was determined by qRTPCR on total NanoDrop ND-1000 (Thermo Scientific, Wilmington, DE) -quantified cellular RNA extracted using RNEasy (Qiagen, Germantown, MD). Qiagen RT kit and SYBR Green PCR Master Mix were used for first-strand synthesis and cDNA amplification, respectively, using a CFX384 cycler (BioRad, Hercules, CA). The primers we used were as follows:

18S forward, 5′ GTAACCCGTTGAACCCCATT-3′;

18S reverse, 5′-CCATCCAATCGGTAGTAGCG-3′;

TCONS_00005559 forward, 5′-AAGGAGAAGAAGGGGAGGAAT-3′;

TCONS_00005559 reverse, 5′-TGCAAATGTTTGGAGAAGACC-3′;

TCONS_00004205 forward, 5′-ACGGGAGAGAGTGACTGGAA-3′;

TCONS_00004205 reverse, 5′-CACCACTTTTGTCCGTGAACT-3′;

LINC01119 forward, 5′-TGGCAAGCTACACTCTGTCA-3′;

LINC01119 reverse, 5′-AGAAGCTGTCCAATGCAACG-3′;

TCONS_00013598 forward, 5′-CATATCACCCAACCTCGCTAA-3′;

TCONS_00013598 reverse, 5′-TGCCATTCAGAGCAGAGAAA-3′;

TCONS_00017736 forward, 5′-TGAGATCATGGAGGAAGTGAA-3′;

TCONS_00017736 reverse, 5′-TTTCGAGGGCTACTGAAGAA-3′;

LINC01133 forward, 5′-GGCAAGGTGAACCTCAAAAA-3′;

LINC01133 reverse, 5′-TTCCTGCAAGAGGAGAAAGC-3′;

TCONS_00019082 forward, 5′-TGTGGAAATGCAGAGAGCAC-3′;

TCONS_00019082 reverse, 5′-GGCACCTGGTGTTTTGTCTT-3′;

LINC01119 iso 1 + 2 forward, 5′-GATGGACCCAGGATGTGAG-3′;

LINC01119 iso 1 + 2 reverse, 5′-GAAAGGGAACACCTCATGGA-3′;

LINC01119 iso 3 forward, 5′-ATGAGATCAGCCACCCTGTC-3′;

LINC01119 iso 3 reverse, 5′-GAAAGGGAACACCTCATGGA-3′;

LINC01119 iso 4 forward, 5′-TCTGCTACTCCCGTGCTTG-3′;

LINC01119 iso 4 reverse, 5′-GAAAGGGAACACCTCATGGA-3′;

GAPDH forward, 5′-ACAACTTTGGTATCGTGGAAGG-3′;

GAPDH reverse, 5′-GCCATCACGCCACAGTTTC-3′;

U1 forward, 5′-CAGGGCGAGGCTTATCCA-3′;

U1 reverse, 5′-GCAGGGGTCAGCACATCC-3′;

SOCS5 forward, 5’-GTGCCACAGAAATCCCTCAAA-3’;

SOCS5 reverse, 5’-TCTCTTCGTGCAAGTCTTGTTC-3’. Target RNA sequence abundance was determined using the 2^−∆∆Ct^ method with 18S employed as a normalization control.

For clinical specimens, qRTPCR measurements were performed on total RNeasy (Qiagen, Hilden, Germany) -purified RNA derived from laser-captured cancer cells macrodissected from specimen slides corresponding to different breast cancer subtypes.

### Constructs

Human LINC01119 sequence (NR_024452; corresponding to ENST00000490950.5) was synthesized by Genscript (Piscataway, NJ) and sub-cloned into pLVX plasmid (provided by P. Pandolfi, Beth Israel Deaconess Medical Center, Boston, MA) using EcoRI and BamHI restriction sites (Forward primer: 5’-TGATTGGAATTCACATAAGTGTGAGTGACTCCT-3’; Reverse primer: 5’-CGCGGA TCCGATTTCACAAATCACATGG-3’). Human SOCS5 CDS sequence (NM_014011) was amplified from MDA-MB-231 cDNA and cloned into pLVX plasmid using EcoRI and BamHI restriction sites using Gibson assembly (NEB, Ipswich, MA) (Forward primer: 5’-gatctcgagctcaagcttcgATGGATAAAGTGGGAAAAATG-3’; Reverse primer: 5’-agaattatctagagtcgcggTTACTTTGCCTTGACTGG-3’). pFLAG-CMV-1, pFLAG-CMV-1-EZH2, and pFLAG-CMV-1-Vimentin plasmids were provided by W. Wei (Beth Israel Deaconess Medical Center, Boston, MA). Human SOCS5 mission shRNA plasmids TRCN0000001895 (#1), TRCN0000001898 (#2), TRCN0000218055 (#3), TRCN0000218597 (#4), and TRCN0000226420 (#5) were obtained from Millipore Sigma (St. Louis, MO).

### Transfections and virus production

For viral particle preparation, HEK293T cells were co-transfected with lentiviral plasmids (e.g., pLVX-LINC01119, pLKO-shSOCS5, or their controls) along with VSVG and psPAX2 using Fugene (Promega, Madison, WI). After 48 h, culture supernatants were collected, passed through 0.45 µm filters, and then added onto target cells in the presence of polybrene (Santa Cruz Biotechnology, Dallas, TX) for 12 h. Stable cell lines were then selected in Puromycin (2 µg/ml; Wisent Bioproducts, Saint-Jean-Baptiste, QC) for ~5 days. For GFP-labeling of MDA-MB-231 cells, pRRL3-GFP lentiviral particles were transduced into recipient cells at two 48-hr intervals, resulting in cells stably expressing GFP at >98% positivity under fluorescence microscopy. For LNC01119 antagonism, LINC01119 ASOs were synthesized by Integrated DNA Technologies (IDT, Coralwille, IA) with the following sequences:

DNA 193T*G*T*C*T*C*T*T*C*C*A*G*C*C*C*A*A*C*A*C;

DNA 791T*A*G*C*T*T*G*C*C*A*C*A*G*C*C*C*A*A*G*C;

DNA 415A*A*G*A*C*C*T*T*G*G*A*G*C*T*C*A*T*C*C*G;

DNA 260A*T*G*G*C*C*C*C*A*G*T*C*C*C*T*T*C*A*G*C;

DNA 893T*G*G*T*C*C*C*T*C*A*A*G*G*C*T*A*G*A*G*G. For control, we used the following sequence: NC5 PS G*C*G*A*C*T*A*T*A*C*G*C*G*C*A*A*T*A*T*G. The esiRNAs targeting RLUC (EHURLUC) or human SOCS5 (EHU109441) were purchased from Millipore Sigma (St. Louis, MO). These ASOs and esiRNA were transfected into target cells using Lipofectamine RNAiMAX Transfection Reagent or Lipofectamine 2000 (Thermo Scientific, Rockford, IL) according to the manufacturer’s protocol.

### Transcription and translation assays

DNA templates for in vitro transcription were generated by PCR of either full-length LINC01119 or of the potential coding sequence #1 of LINC01119 (232 bp) from the pLVX-LINC01119 plasmid template amplified using T7-promoter-inserting and Kozak-inserting primer sequences and reverse primers annealing to the poly-A tail. In vitro translation was conducted with Transcend non-radioactive translation detection system (L1170, Promega, Madison, WI). For *in-cell* translation of full-length or potential LINC01119 ORFs, sequences were expressed from pFLAG-CMV-1 plasmid transfected into HEK293T cells. qRTPCR was used to ensure expression efficiency after 48 h, followed by Western blots at 72 h to detect protein products. For *in-cell* translation analyses of LINC01119 potential coding sequence (PCS), FLAG sequence was inserted 3’ of LINC01119 PCSs using QuikChange kit (200555) according to manufacturer’s instruction (Agilent, Santa Clara, CA) using the following primers:

LINC01119-PCS#1 Forward: 5’-CCATGAGGTGTTCCCTTTCGGAGACTACAAAGACGATGACGACAAGTGAGCTCCAAGG-3’.

LINC01119-PCS#1 Reverse: 5’-CCTTGGAGCTCACTTGTCGTCATCGTCTTTGTAGTCTCCGAAAGGGAACACCTCATGG-3’;

LINC01119-PCS#4 Forward: 5’-GTCTGAACGACGGTCCGACTACAAAGACGATGACGACAAGTGAGCAAGAACCACCT-3’,

LINC01119-PCS#4 Reverse: 5’-AGGTGGTTCTTGCTCACTTGTCGTCATCGTCTTTGTAGTCGGACCGTCGTTCAGAC-3’. Constructs were then transfected into HEK293T cells. Lysates were recovered after 72 h and FLAG-tagged products detected by Western blotting using FLAG (#14793, Cell Signaling Technology, Danvers, MA) and β-actin (#4970, CST) antibodies.

### RNAscope

RNAscope-based in situ hybridization for LINC01119 was performed using ACD HybEZ™ II Oven with RNAscope 2.5 HD detection reagent-Brown according to manufacturer’s protocols (Advanced Cell Diagnostics, Hayward, CA) with 18 ZZ probe pairs synthesized against sequence 29-957 of NCBI accession number NR_024452.1 for human-LINC01119 (#537331, ACD). Probes for human peptidylprolyl isomerase B (PPIB) and for bacterial dapB were used as positive and negative controls, respectively.

### Proliferation, cell cycle, anchorage-independent growth, suspension, and colony-formation assays

For cell proliferation assays, cancer cells were seeded in quadruplicates in 12-well plates (25.0 × 10^3^ per well) and counted using the Trypan blue exclusion assay every day for 4 days. Alternatively, cell growth was monitored in cancer cells seeded into 96-well plates (5.0 × 10^3^ per well), and growth was estimated using WST-1 (Millipore Sigma, St. Louis, MO) or CellTiter 96 (G3580, Promega, Madison, WI) at the indicated days. For cell cycle analyses, 1 × 10^6^ cold-PBS-washed cells were suspended in 500 µl PI/Triton X-100 staining solution. Data acquisition was performed using flow cytometry and analyzed by ModFit LT (Macintosh). For anchorage-independent growth, cancer cells were mixed with equal volumes of 0.35% agar and seeded into pre-coated (0.625% agar) in 6-well dishes at a density of 5.0 × 10^3^ cells per well. Colonies were stained with 0.002% Crystal Violet after 3 weeks and colony counts estimated using ImageJ software (NIH Image). For suspension assays, a total of 5.0 × 10^3^ cancer cells were suspended in 1.7 ml Eppendorf tubes containing 1.5 ml DMEM medium with 0.1% FBS under constant tumbling rotation. Live cells were counted using the Trypan blue exclusion assay every 24 h for the duration of the experiment. For low-density adherent colony-formation cultures, cancer cells (500 cells) were plated in 6 cm dishes, fed and maintained for 2 weeks, then growths fixed with 100% methanol for 20 mins. Colonies were stained with Crystal Violet and counted using ImageJ software (NIH Image).

### Western blotting

Western blots were performed using standard techniques with 1:1000 dilutions of antibodies from Cell Signaling Technology (CST; Danvers, MA): FLAG (#14793), β-actin (#4970), GAPDH (#2118), JAK1 (#3344), p-JAK1 (Y1034/1035) (#74129), JAK2 (#3230), p-JAK2 (Y1008) (#8082), JAK3 (#8827), p-JAK3 (Y980/981) (#5031), TYK2 (#14193), p-TYK2 (Y1054/1055) (#68790), STAT1 (#14994), p-STAT1 (Tyr 701) (#7649), STAT2 (#72604), p-STAT2 (Tyr690) (#4441), STAT3 (#30835), p-STAT3 (Tyr705) (#9145), STAT4 (#2653), p-STAT4 (Tyr693) (#4134), STAT5 (#94205), p-STAT5 (Tyr694) (#4322), STAT6 (#5397), and p-STAT6 (Tyr641) (#9361). Antibodies from Sigma (St. Louis, MO) included SOCS5 (WH0009655M1) (Dilution 1:1000), and Vinculin (#V9131) (Dilution 1:1000). Blots were developed using chemiluminescence (BioRad, Hercules, CA). All blots derive from the same experiment and were processed in parallel.

### Immunohistochemistry (IHC)

IHC was performed using standard techniques. Nuclei with any detectable Ki67 antibody (EPR3610; ab92742; Abcam; 1:200 dilution) staining above background levels (negative control without primary antibody) were scored as positive cells. Positive staining was scored blindly in at least five random fields per slide at ×200 magnification.

### Tumorigenesis assay

Animal experiments were performed according to approved procedures of the BIDMC’s Institutional Animal Care and Use Committee. For LINC01119, female NCG mice (NOD-Prkdc^em26Cd52^IL2rg^em26Cd22^/NjuCrl Coisogenic Immunodeficient) (6 weeks old; Charles River, MA) were injected orthotopically with 2.5 × 10^5^ cells (SUM159-pLVX or SUM159-pLVX-LINC01119) in 1:1 DMEM: Matrigel suspension into mammary fat pads. For hSOCS5, female Nu/Nu mice (5 weeks old; Charles River, MA) were injected orthotopically with 2.5 × 10^5^ cells (SUM159-pLVX or SUM159-pLVX-SOCS5) in 1:1 DMEM: Matrigel suspension into mammary fat pads. NCG and Nu/Nu mice were sacrificed 42 and 46 days post-injection, respectively, and tumors were surgically excised and weighed.

### Clinical analyses

Association of LINC01119 with clinical breast cancer subtypes was derived from TANRIC database (ibl.mdanderson.org) on TCGA data (LINC01119 was queried by position chr2:47055003-47086145). LINC01119 expression levels in laser-captured tissues were estimated using qRTPCR on Qiagen-purified RNA extracted from cancer cells lifted off BLBC, HER2, luminal A, and luminal B tissue slides prepared at the Curie Institute and derived from human specimens collected in compliance with ethical regulations, informed patient consent, and approval of the Curie IRB. LINC01119 and SOCS5 expression levels in clinical breast cancer specimens were obtained from GENT2 database^71^. STAT6 expression levels in clinical breast cancer specimens were obtained from UALCAN (http://ualcan.path.uab.edu) that included subclass and TP53 mutation status. For data display, we chose the box-and-whisker plots, in which the median is represented by the centerline. The whiskers extend up to 1.5 times the Inter Quartile Range from the box to the smallest and largest points. Correlation analyses between LINC01119 (probe: 230799_at) and SOCS5 (probe: 209648_x_at) were performed using breast cancer data sets in CCLE (GSE36133), GSE28844, GSE16446, GSE102484, GSE12276, GSE76124, and GSE27830 all derived from the GEO database. Correlation analyses between SOCS5 (probe: 209648_x_at) and STAT6 (probe: 201331_s_at) were performed using R2 data (https://hgserver1.amc.nl/cgi-bin/r2/main.cgi). For patient survival curves, median-centered log ratios of both LINC01119 and SOCS5 (or STAT6) were partitioned into high-expression and low-expression specimen groups, and analyses were conducted using the log-rank test and the proportional hazard model to compare KM survival curves.

### Statistical analyses

A two-sided unpaired Student’s *t*-test was used to analyze the significance between means ± SD of at least three independent biological replicates. One-way ANOVA followed by Tukey posthoc test was performed when appropriate to analyze the differences between individual experiments in multiple comparisons. Bivariate Pearson correlation (SPSS: version 23) was used to test LINC01119 and SOCS5 associations. For all analyses, *, **, and *** indicated *p* < 0.05, *p* < 0.01, and *p* < 0.001, respectively.

### Reporting summary

Further information on research design is available in the Nature Research Reporting Summary linked to this article.

## Supplementary information

Supplementary Information

Reporting Summary

## Data Availability

The data generated and analyzed during this study are described in the following data record: 10.6084/m9.figshare.14377130^72^. The RNA sequencing data are openly available in the Gene Expression Omnibus via the following accession: GSE171121^73^. The data underlying the figures and supplementary figures of the related article are openly available via the figshare repository at 10.6084/m9.figshare.14327099.v1^74^. Requests for plasmids and other reagents should be addressed to the corresponding author.

## Code availability

No custom codes or scripts were used in the generation or analysis of presented datasets.

## References

[1] Bray F (2018). Global cancer statistics 2018: GLOBOCAN estimates of incidence and mortality worldwide for 36 cancers in 185 countries. CA Cancer J. Clin..

[2] Waks AG, Winer EP (2019). Breast cancer treatment: a review. JAMA.

[3] Perou CM (2000). Molecular portraits of human breast tumours. Nature.

[4] Sorlie T (2001). Gene expression patterns of breast carcinomas distinguish tumor subclasses with clinical implications. Proc. Natl Acad. Sci. USA.

[5] Curtis C (2012). The genomic and transcriptomic architecture of 2,000 breast tumours reveals novel subgroups. Nature.

[6] Cancer Genome Atlas, N. (2012). Comprehensive molecular portraits of human breast tumours. Nature.

[7] Howlader, N. et al (2014). US incidence of breast cancer subtypes defined by joint hormone receptor and HER2 status. J. Natl. Cancer Inst.

[8] Bardia A (2017). Efficacy and safety of anti-trop-2 antibody drug conjugate Sacituzumab Govitecan (IMMU-132) in heavily pretreated patients with metastatic triple-negative breast cancer. J. Clin. Oncol..

[9] Swain SM (2015). Pertuzumab, trastuzumab, and docetaxel in HER2-positive metastatic breast cancer. N. Engl. J. Med..

[10] Carey L, Winer E, Viale G, Cameron D, Gianni L (2010). Triple-negative breast cancer: disease entity or title of convenience?. Nat. Rev. Clin. Oncol..

[11] Zhang XH (2013). Selection of bone metastasis seeds by mesenchymal signals in the primary tumor stroma. Cell.

[12] Chaturvedi P, Gilkes DM, Takano N, Semenza GL (2014). Hypoxia-inducible factor-dependent signaling between triple-negative breast cancer cells and mesenchymal stem cells promotes macrophage recruitment. Proc. Natl Acad. Sci. USA.

[13] Karnoub AE (2007). Mesenchymal stem cells within tumour stroma promote breast cancer metastasis. Nature.

[14] Karnoub, A. E. Multifunctional roles of tumor-associated mesenchymal stem cells in cancer progression. In *Mesenchymal Stromal Cells as Tumor Stromal Modulators*. (eds. M. Bolontrade & M. Garcia) 335–360 (Academic Press, 2016).

[15] El-Haibi CP (2012). Critical role for lysyl oxidase in mesenchymal stem cell-driven breast cancer malignancy. Proc. Natl Acad. Sci. USA.

[16] Cuiffo BG (2014). MSC-regulated microRNAs converge on the transcription factor FOXP2 and promote breast cancer metastasis. Cell Stem Cell.

[17] Houthuijzen JM, Daenen LG, Roodhart JM, Voest EE (2012). The role of mesenchymal stem cells in anti-cancer drug resistance and tumour progression. Br. J. Cancer.

[18] Schmitt AM, Chang HY (2016). Long noncoding RNAs in cancer pathways. Cancer Cell.

[19] Anastasiadou, E., Faggioni, A., Trivedi, P. & Slack, F. J. The nefarious nexus of noncoding RNAs in cancer. *Int. J. Mol. Sci.* **19**, 2072 (2018). 10.3390/ijms19072072 PMC6073630 30018188

[20] Brown, J. M., Wasson, M. D. & Marcato, P. The Missing Lnc: the potential of targeting triple-negative breast cancer and cancer stem cells by inhibiting long non-coding RNAs. *Cells* **9**, 763 (2020). 10.3390/cells9030763 PMC7140662 32244924

[21] Tu, Z., Schmollerl, J., Cuiffo, B. G. & Karnoub, A. E. Microenvironmental regulation of long noncoding RNA LINC01133 promotes cancer stem cell-like phenotypic traits in triple-negative breast cancers. Stem Cells 37, 1281–1292 (2019). 10.1002/stem.3055 31283068

[22] He W (2018). Long noncoding RNA BLACAT2 promotes bladder cancer-associated lymphangiogenesis and lymphatic metastasis. J. Clin. Invest..

[23] Lan X (2016). A novel long noncoding RNA Lnc-HC binds hnRNPA2B1 to regulate expressions of Cyp7a1 and Abca1 in hepatocytic cholesterol metabolism. Hepatology.

[24] Drews-Elger K (2014). Primary breast tumor-derived cellular models: characterization of tumorigenic, metastatic, and cancer-associated fibroblasts in dissociated tumor (DT) cultures. Breast Cancer Res. Treat..

[25] Matsumoto A (2017). mTORC1 and muscle regeneration are regulated by the LINC00961-encoded SPAR polypeptide. Nature.

[26] Huang JZ (2017). A peptide encoded by a putative lncRNA HOXB-AS3 suppresses colon cancer growth. Mol. Cell.

[27] Zeng C, Fukunaga T, Hamada M (2018). Identification and analysis of ribosome-associated lncRNAs using ribosome profiling data. BMC Genomics.

[28] Tripathi V (2010). The nuclear-retained noncoding RNA MALAT1 regulates alternative splicing by modulating SR splicing factor phosphorylation. Mol. Cell.

[29] Mello SS (2017). Neat1 is a p53-inducible lincRNA essential for transformation suppression. Genes Dev..

[30] Lin A (2016). The LINK-A lncRNA activates normoxic HIF1alpha signalling in triple-negative breast cancer. Nat. Cell Biol..

[31] Jiang Z (2017). LincIN, a novel NF90-binding long non-coding RNA, is overexpressed in advanced breast tumors and involved in metastasis. Breast Cancer Res..

[32] Cooney RN (2002). Suppressors of cytokine signaling (SOCS): inhibitors of the JAK/STAT pathway. Shock.

[33] Hu, H. et al. MicoRNA-301a promotes pancreatic cancer invasion and metastasis through the JAK/STAT3 signaling pathway by targeting SOCS5. *Carcinogenesis* **41**, 502–514 (2019). 10.1093/carcin/bgz121 31233116

[34] Sharma ND (2019). Epigenetic silencing of SOCS5 potentiates JAK-STAT signaling and progression of T-cell acute lymphoblastic leukemia. Cancer Sci..

[35] Zhang J (2020). miR-101 inhibits feline herpesvirus 1 replication by targeting cellular suppressor of cytokine signaling 5 (SOCS5). Vet. Microbiol..

[36] Linossi EM (2013). Suppressor of Cytokine Signaling (SOCS) 5 utilises distinct domains for regulation of JAK1 and interaction with the adaptor protein Shc-1. PLoS ONE.

[37] Chandrashekaran IR (2015). Structure and functional characterization of the conserved jak interaction region in the intrinsically disordered N-terminus of SOCS5. Biochemistry.

[38] Linossi EM, Nicholson SE (2015). Kinase inhibition, competitive binding and proteasomal degradation: resolving the molecular function of the suppressor of cytokine signaling (SOCS) proteins. Immunol. Rev..

[39] Toniolo PA (2016). Deregulation of SOCS5 suppresses dendritic cell function in chronic lymphocytic leukemia. Oncotarget.

[40] Seki Y (2002). Expression of the suppressor of cytokine signaling-5 (SOCS5) negatively regulates IL-4-dependent STAT6 activation and Th2 differentiation. Proc. Natl Acad. Sci. USA.

[41] Burstein MD (2015). Comprehensive genomic analysis identifies novel subtypes and targets of triple-negative breast cancer. Clin. Cancer Res..

[42] Argos M (2008). Genomewide scan for loss of heterozygosity and chromosomal amplification in breast carcinoma using single-nucleotide polymorphism arrays. Cancer Genet. Cytogenet..

[43] O’Connell P (1998). Analysis of loss of heterozygosity in 399 premalignant breast lesions at 15 genetic loci. J. Natl Cancer Inst..

[44] Smith P (2006). A genome wide linkage search for breast cancer susceptibility genes. Genes Chromosomes Cancer.

[45] Samuelson E, Levan K, Adamovic T, Levan G, Horvath G (2008). Recurrent gene amplifications in human type I endometrial adenocarcinoma detected by fluorescence in situ hybridization. Cancer Genet Cytogenet..

[46] Purdue MP (2011). Genome-wide association study of renal cell carcinoma identifies two susceptibility loci on 2p21 and 11q13.3. Nat. Genet..

[47] Okhrimenko H, Lu W, Xiang C, Hamburger N, Kazimirsky G, Brodie C. Protein kinase C-epsilon regulates the apoptosis and survival of glioma cells. Cancer Res. 2005 Aug 15;65(16):7301-9. doi: 10.1158/0008-5472.CAN-05-1064. PMID: 16103081; PMCID: PMC1360842. 10.1158/0008-5472.CAN-05-1064 PMC1360842 16103081

[48] Chen H, Wu X, Pan ZK, Huang S (2010). Integrity of SOS1/EPS8/ABI1 tri-complex determines ovarian cancer metastasis. Cancer Res..

[49] Engreitz JM (2016). Local regulation of gene expression by lncRNA promoters, transcription and splicing. Nature.

[50] Seashols-Williams SJ (2016). miR-9 acts as an OncomiR in prostate cancer through multiple pathways that drive tumour progression and metastasis. PLoS ONE.

[51] Su M, Qin B, Liu F, Chen Y, Zhang R (2018). miR-885-5p upregulation promotes colorectal cancer cell proliferation and migration by targeting suppressor of cytokine signaling. Oncol. Lett..

[52] Sanchez-Mejias A (2019). A novel SOCS5/miR-18/miR-25 axis promotes tumorigenesis in liver cancer. Int. J. Cancer.

[53] Long J (2018). Maintenance of stemness by miR-589-5p in hepatocellular carcinoma cells promotes chemoresistance via STAT3 signaling. Cancer Lett..

[54] Yan D, Jin F, Lin Y (2020). lncRNA HAND2-AS1 inhibits liver cancer cell proliferation and migration by upregulating SOCS5 to inactivate the JAK-STAT pathway. Cancer Biother. Radiopharm..

[55] Bi, H. Q., Li, Z. H. & Zhang, H. Long noncoding RNA HAND2-AS1 reduced the viability of hepatocellular carcinoma via targeting microRNA-300/SOCS5 axis. *Hepatobiliary Pancreat Dis. Int.* **19**, 567–574 (2020). 10.1016/j.hbpd.2020.02.011 32224127

[56] Ye F, Tian L, Zhou Q, Feng D (2019). LncRNA FER1L4 induces apoptosis and suppresses EMT and the activation of PI3K/AKT pathway in osteosarcoma cells via inhibiting miR-18a-5p to promote SOCS5. Gene.

[57] Wu X, Cai D, Zhang F, Li M, Wan Q (2019). Long noncoding RNA TUSC7 inhibits cell proliferation, migration and invasion by regulating SOCS4 (SOCS5) expression through targeting miR-616 in endometrial carcinoma. Life Sci..

[58] Tan J, Xiang L, Xu G (2019). LncRNA MEG3 suppresses migration and promotes apoptosis by sponging miR-548d-3p to modulate JAK-STAT pathway in oral squamous cell carcinoma. IUBMB Life.

[59] Liu, Z. et al. LINC00668 modulates SOCS5 expression through competitively sponging miR-518c-3p to facilitate glioma cell proliferation. *Neurochem. Res.* 45, 1614–1625 (2020). 10.1007/s11064-020-02988-2 32279214

[60] Zhang Z (2019). BRM transcriptionally regulates miR-302a-3p to target SOCS5/STAT3 signaling axis to potentiate pancreatic cancer metastasis. Cancer Lett..

[61] Nicholson SE (2005). Suppressor of cytokine signaling (SOCS)-5 is a potential negative regulator of epidermal growth factor signaling. Proc. Natl Acad. Sci. USA.

[62] Kario E (2005). Suppressors of cytokine signaling 4 and 5 regulate epidermal growth factor receptor signaling. J. Biol. Chem..

[63] Avraham R, Yarden Y (2011). Feedback regulation of EGFR signalling: decision making by early and delayed loops. Nat. Rev. Mol. Cell Biol..

[64] Durham GA, Williams JJL, Nasim MT, Palmer TM (2019). Targeting SOCS proteins to control JAK-STAT signalling in disease. Trends Pharm. Sci..

[65] Hong X (2014). Opposing activities of the Ras and Hippo pathways converge on regulation of YAP protein turnover. EMBO J..

[66] Evans MK (2007). Expression of SOCS1 and SOCS3 genes is differentially regulated in breast cancer cells in response to proinflammatory cytokine and growth factor signals. Oncogene.

[67] Liu C, Li W, Zhang L, Song C, Yu H (2019). Tumor-suppressor microRNA-151-5p regulates the growth, migration and invasion of human breast cancer cells by inhibiting SCOS5. Am. J. Transl. Res..

[68] Zhang M (2019). SOCS5 inhibition induces autophagy to impair metastasis in hepatocellular carcinoma cells via the PI3K/Akt/mTOR pathway. Cell Death Dis..

[69] Papageorgis P (2015). Targeting IL13Ralpha2 activates STAT6-TP63 pathway to suppress breast cancer lung metastasis. Breast Cancer Res..

[70] DiScala M (2020). Loss of STAT6 leads to anchorage-independent growth and trastuzumab resistance in HER2+ breast cancer cells. PLoS ONE.

[71] Park SJ, Yoon BH, Kim SK, Kim SY (2019). GENT2: an updated gene expression database for normal and tumor tissues. BMC Med. Genomics.

[72] Tu, Z. et al. Metadata record for the manuscript: the LINC01119-SOCS5 axis as a critical theranostic in triple-negative breast cancer. *figshare*10.6084/m9.figshare.14377130 (2021). 10.1038/s41523-021-00259-z PMC8166834 34059683

[73] Tu, Z. & Karnoub, A. E. MSC-induced lncRNA expression changes in breast cancer cells. *Gene Expression Omnibus* https://identifiers.org/geo:GSE171121 (2021).

[74] Tu, Z. & Karnoub, A. E. The LINC01119-SOCS5 axis as a critical theranostic in triple-negative breast cancer. *figshare*10.6084/m9.figshare.14327099.v1 (2021). 10.1038/s41523-021-00259-z PMC8166834 34059683

