# Supplementary material for: The LINC01119-SOCS5 axis as a critical theranostic in triple-negative breast cancer
Source: NPJ Breast Cancer. 2021 May 31;7:69. doi: 10.1038/s41523-021-00259-z (PMC8166834; doi:10.1038/s41523-021-00259-z)

a

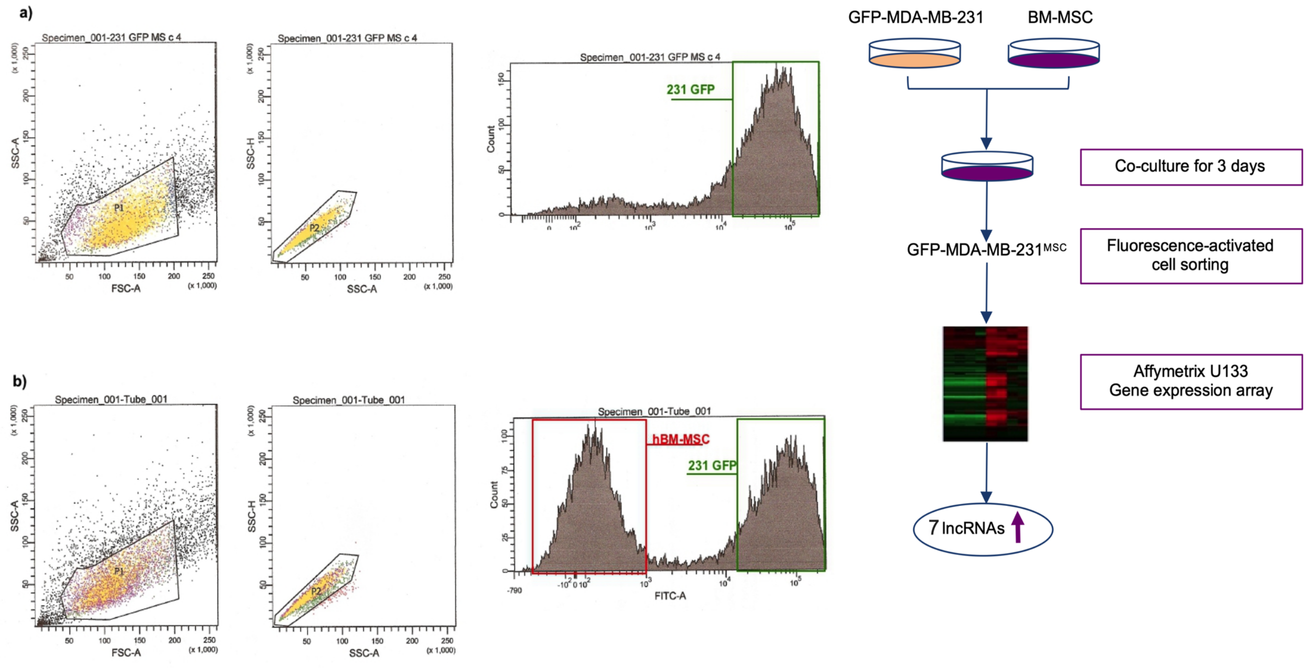

b

Transcript ID	Fold Change	q-value(%)
TCONS_00005559	4.000	0.000
TCONS_00004205	2.166	0.000
TCONS_00002647 (LINC01119)	1.903	2.317
TCONS_00013598	1.763	3.014
TCONS_00017736	1.631	2.317
TCONS_l2_00000659 (LINC01133)	1.625	8.664
TCONS_00019082	1.543	0.000

Supplementary Figure 1. *LncRNA induction in MSC-activated cancer cells.* (**a**) Sorting of GFP-labeled MDA-MB-231 cells cultured alone (top row) or together (bottom row) with human BM-MSCs for 72 hrs. Gating shown distinguished GFP-positive cells from debris and cell aggregates. Only the strongest GFP-expressing cells were selected to avoid contamination from GFP-negative MSCs. (**b**) List of lncRNAs induced in MSC-stimulated MDA-MB-231 cells versus control MDA-MB-231 cells cultured alone ranked by fold change. The q-value threshold (false-discovery rate (FDR) adjusted p-value) was set less than 10%; only significant hits were considered in the analyses.

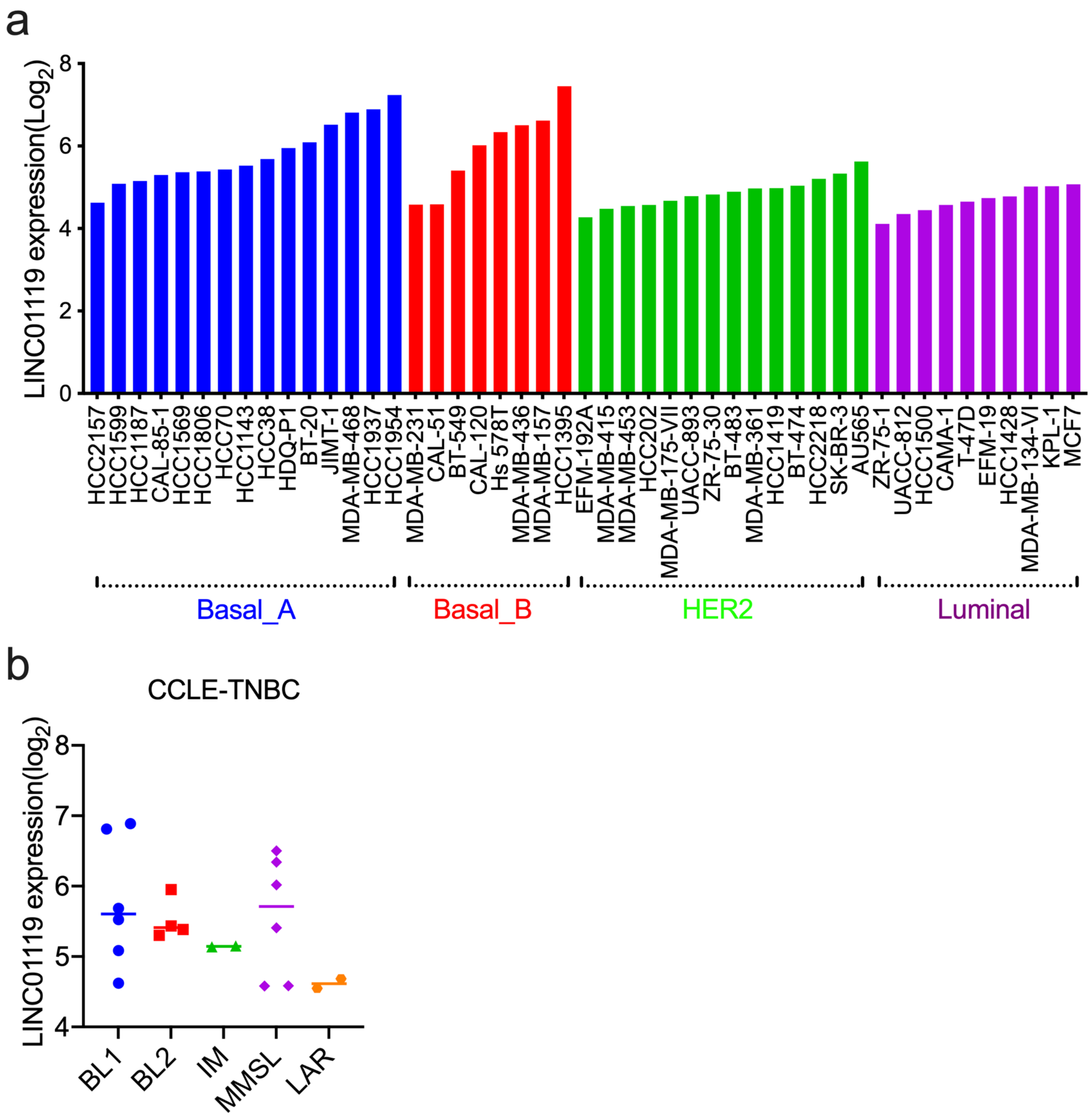

Supplementary Figure 2. *LINC01119 levels across breast cancer cell lines in CCLE*. (**a-b**) LINC01119 levels (probe: 230799_at) in 47 breast cancer cell lines (a) and in 20 classified TNBC cells lines from the Cancer Cell Line Encyclopedia (b).

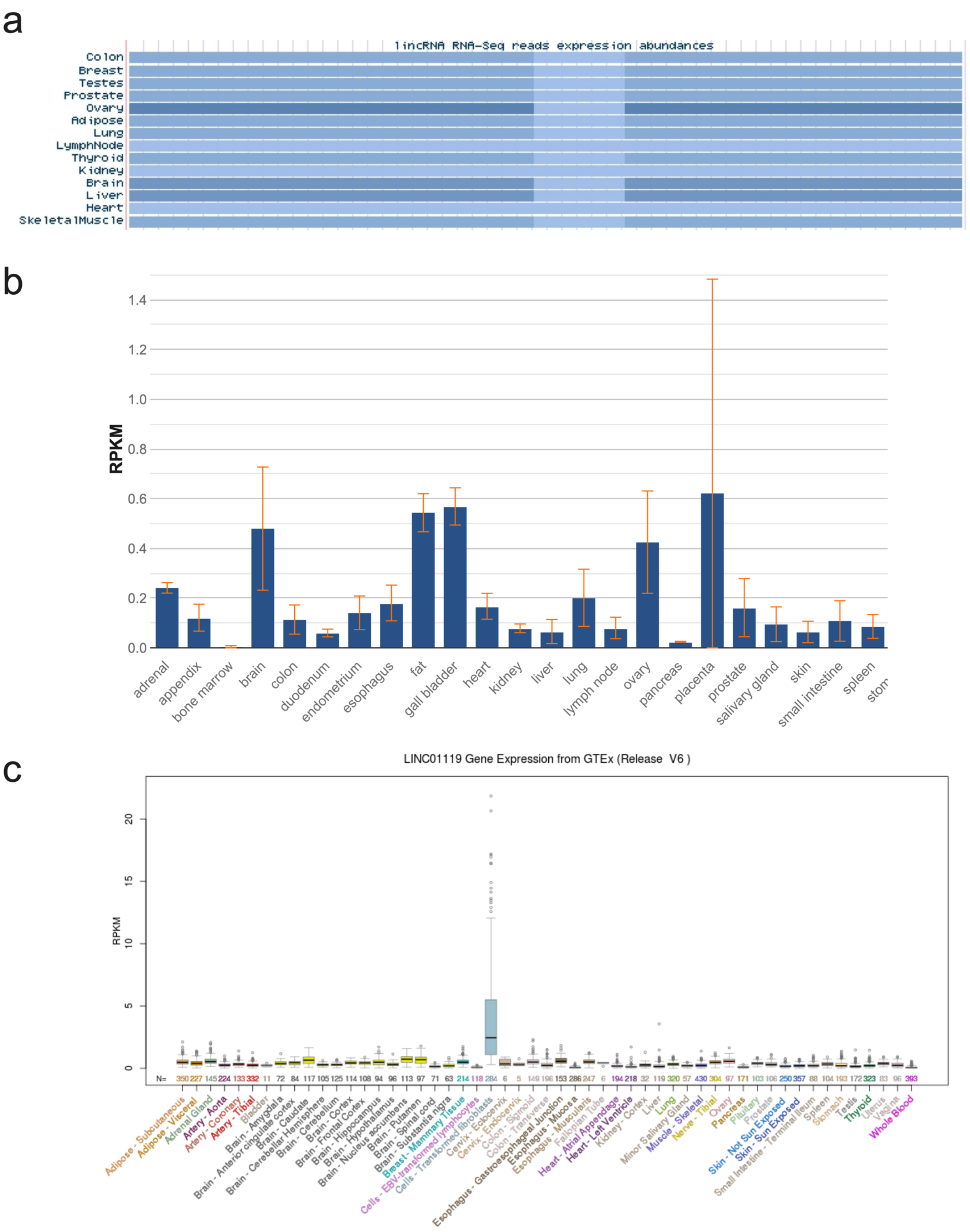

Supplementary Figure 3. *LINC01119 tissue expression patterns*. (**a**) RNA-Seq-based expression abundance of LINC01119 in indicated tissues (UCSC Genome Browser). (**b**) Expression profile (FPKM) of LINC01119 derived from RNA-Seq of multiple tissues in NONCODE. (**c**) Expression profile (FPKM) of LINC01119 in indicated tissues from GTEx.

a

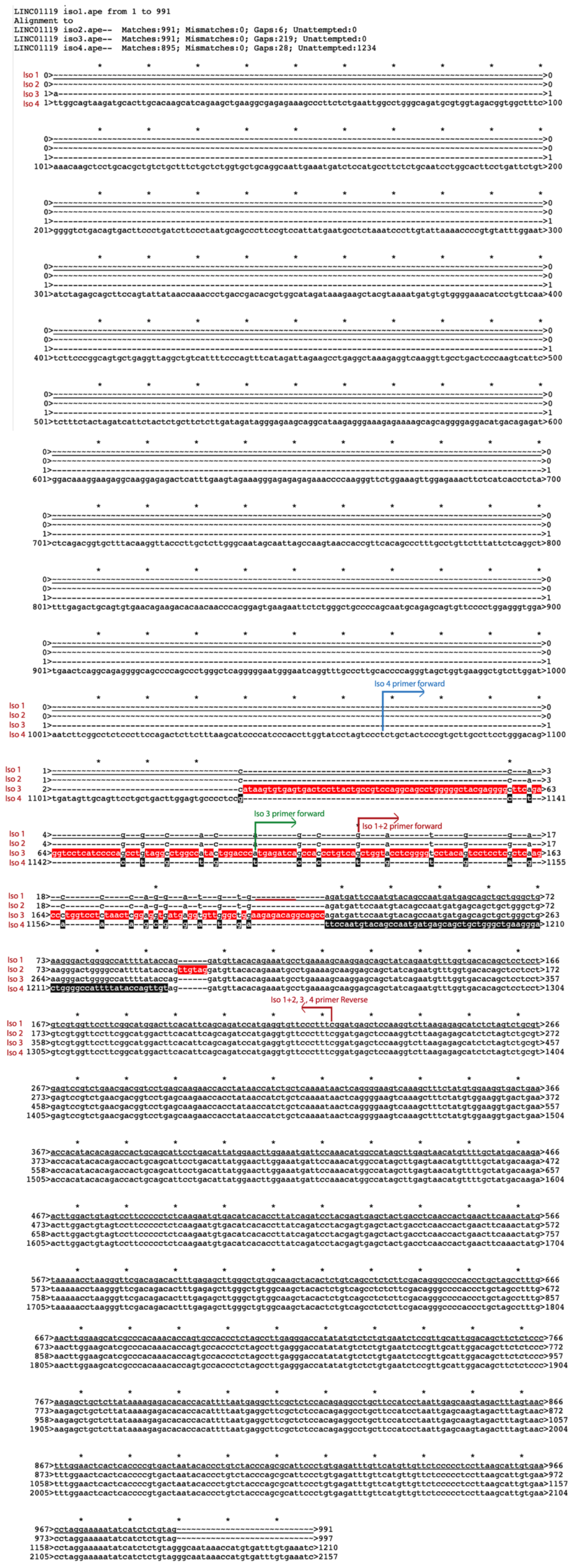

Supplementary Figure 4. *LINC01119 primer design*. (**a**) Schema of specific qRTPCR primers designed to amplify LINC01119 isoforms 1/2, 3 and 4.

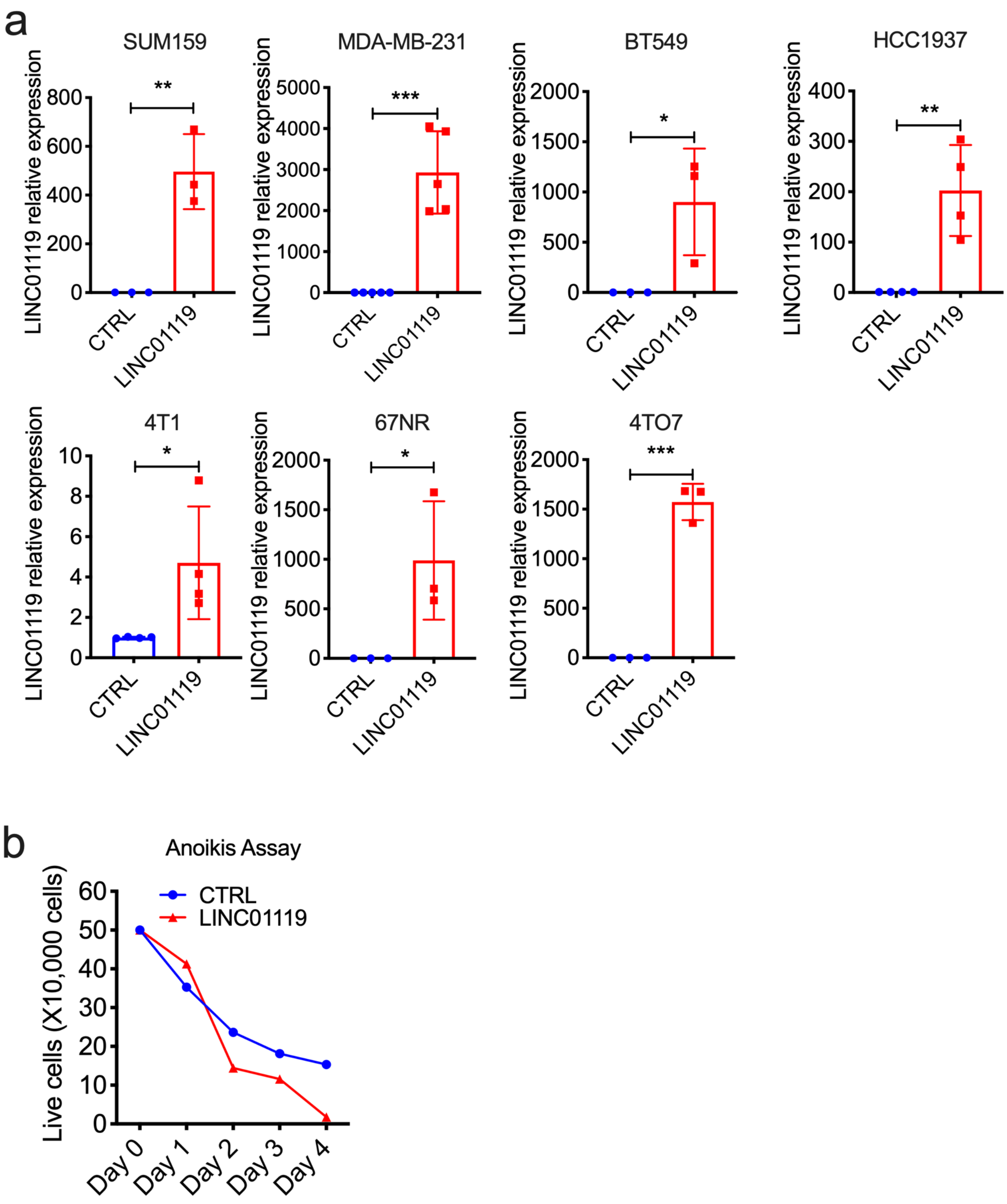

Supplementary Figure 5. *LINC01119 over-expression efficiency and its effects on anoikis*. (**a**) Over-expression efficiency of LINC01119 in SUM159, MDA-MB-231, BT549, HCC1937, 4T1, 67NR and 4T07 cells (mean±SD of n≥3). (**b**) Suspension assay on indicated MDA-MB-231 cell groups with Trypan-blue-positive cells counted at indicated days (mean of n=3 of triplicates).

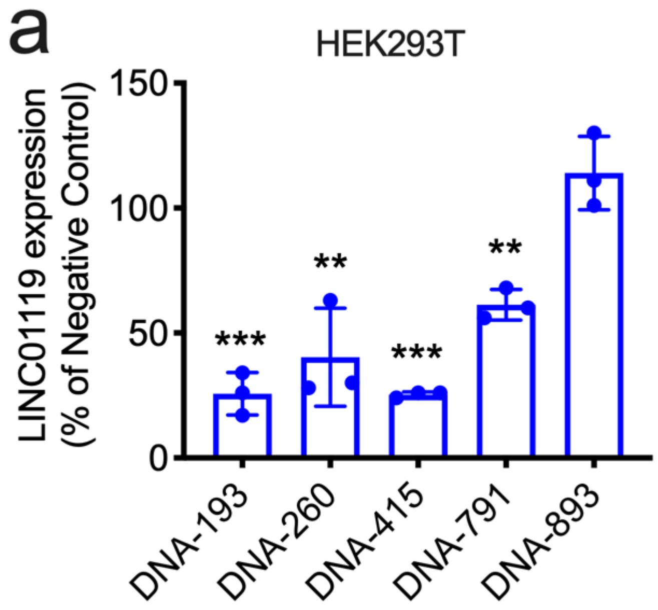

Supplementary Figure 6. *Potency of anti-sense-oligonucleotide-mediated LINC01119 knockdown*. (**a**) qRTPCR (mean±SD of n=3) of the knockdown efficiency of LINC01119 ASOs in HEK293T cells.

a

Cancer	Position/Annotation	Gene Symbol	Correlation	P-value
TCGA-BRCA	2:47055003-47086145	LOC100134259	0.932243	0
TCGA-BRCA	2:47055003-47086145	SOCS5	0.5700105	0
TCGA-BRCA	2:47055003-47086145	ANXA1	0.5658201	0
TCGA-BRCA	2:47055003-47086145	PLAGL1	0.5510195	0
TCGA-BRCA	2:47055003-47086145	BOC	0.5407121	0
TCGA-BRCA	2:47055003-47086145	RBMS1	0.5330831	0

b

genes	R value	P value
SOCS5	0.456	0.0288
ANXA1	0.1062	0.6296
PLAGL1	0.0031	0.9888
BOC	-0.3626	0.0891
RBMS1	-0.1801	0.4109

c d

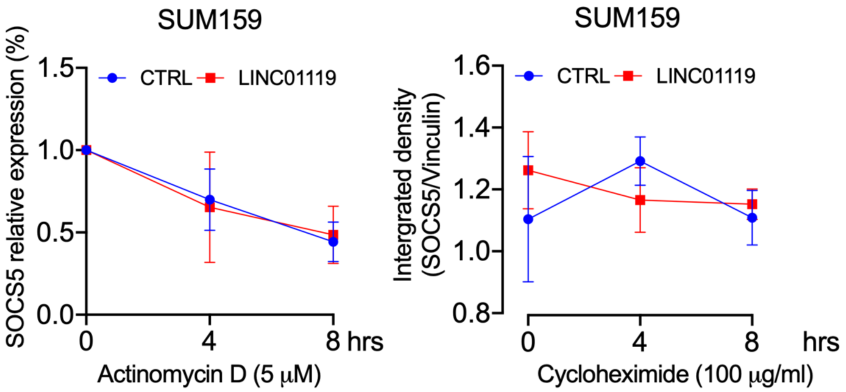

Supplementary Figure 7. *Top LINC01119-correlated genes and the mRNA and protein stability of SOCS5*. (**a**) Top LINC01119-correlated genes from TANRIC. (**b**) Top five commonly correlated genes from TANRIC and TNBC CCLE data. (**c**) qRTPCR (mean±SD of n=3) of *SOCS5* relative expression in control SUM159 cells or counterparts stably over-expressing LINC01119 at indicated time points after treatment with actinomycin D at 0 hrs. 18S was used as a control. (**d**) Relative SOCS5 protein expression (mean±SD of n=3) in control SUM159 cells or counterparts stably over-expressing LINC01119 at indicated time points after treatment with cycloheximide at 0 hrs. Levels were estimated by ImageJ on Western blots using vinculin as a control.

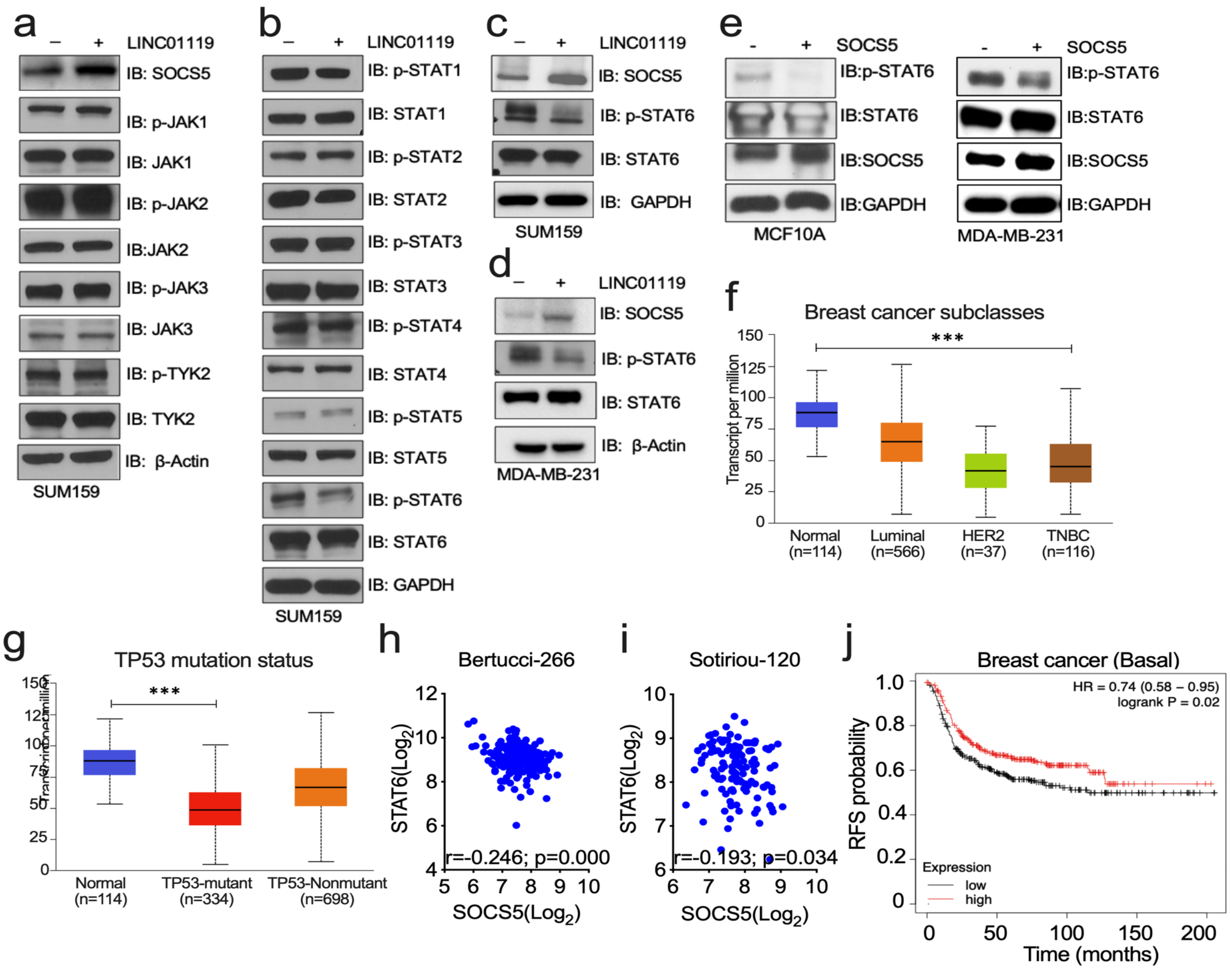

Supplementary Figure 8. *LINC01119-SOCS5 axis inhibits STAT6*. (**a-b**) Representative Western blots (n>3) of JAK family members (a) and STAT family members (b) in SUM159 cells stably expressing LINC01119 after serum-starvation for 16 hrs. (**c-d**) Representative Western blots of phospho-STAT6 in SUM159 cells (n=3) (c) and MDA-MB-231 cells (n=3) (d) stably expressing LINC01119 grown in the presence of serum. (**e**) Representative Western blots of phospho-STAT6 in serum-starved MCF10A cells (n=3) and MDA-MB-231 cells (n=3) 24 hrs after transient transfection with control or SOCS5-expresing vectors. (**f-g**) *STAT6* expression in breast cancer subtypes (f) and in TP53 mutant samples (g) based on UALCAN data. (**h-i**) Correlation of LINC01119 and *SOCS5* gene expression levels in Bertucci-266 (h) and Sotiriou-120 (i). Data derived from R2. (**j**) Kaplan-Meier analysis (kmplot.com) of relapse-free survival (RFS) based on the mean value of *SOCS5* and *STAT6* in basal breast cancer with cutoff as mean value of probe 209648_x_at for *SOCS5* and probe 201331_s_at for *STAT6*.

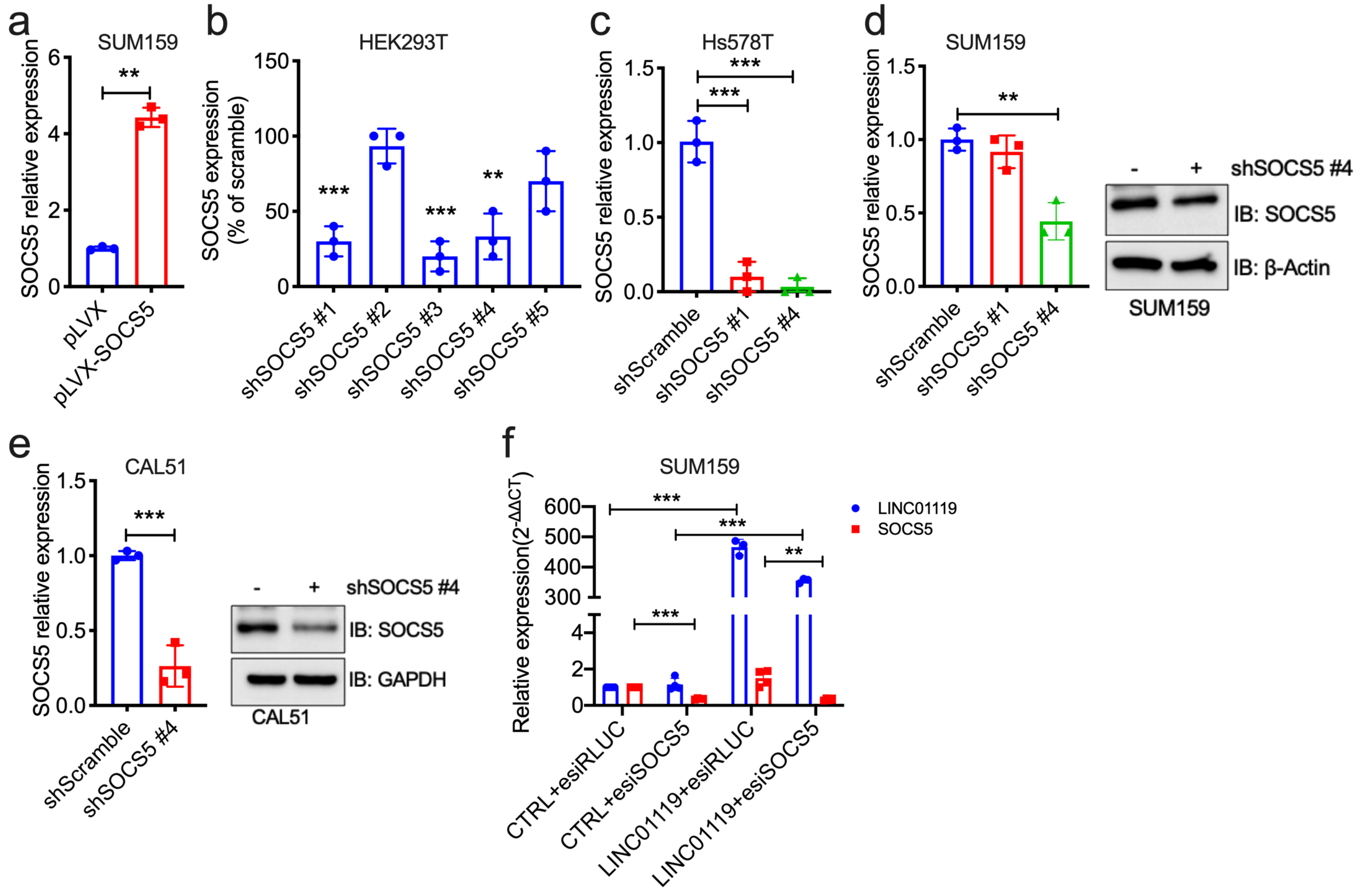

Supplementary Figure 9. *LINC01119 ASOs and SOCS5 shRNA screens*. (**a**) qRTPCR measurements (mean±SD of n=3) of the over-expression efficiency of human SOCS5 in SUM159 cells. (**b-c**) qRTPCR measurements (mean±SD of n=3) of the knockdown efficiency of SOCS5-shRNA in HEK293T cells (b) and Hs578T cells (c). (**d-e**) qRTPCR measurements (mean±SD of n=3) (left) and Western Blot (right) of the knockdown efficiency of SOCS5 in SUM159 cells (d) and CAL51 cells (e). (**f**) qRTPCR measurements (mean±SD of n=3) of the knockdown efficiency of esiRNA-SOCS5 compared to esiRLUC controls in control and LINC01119 over-expressing SUM159 cells.

a

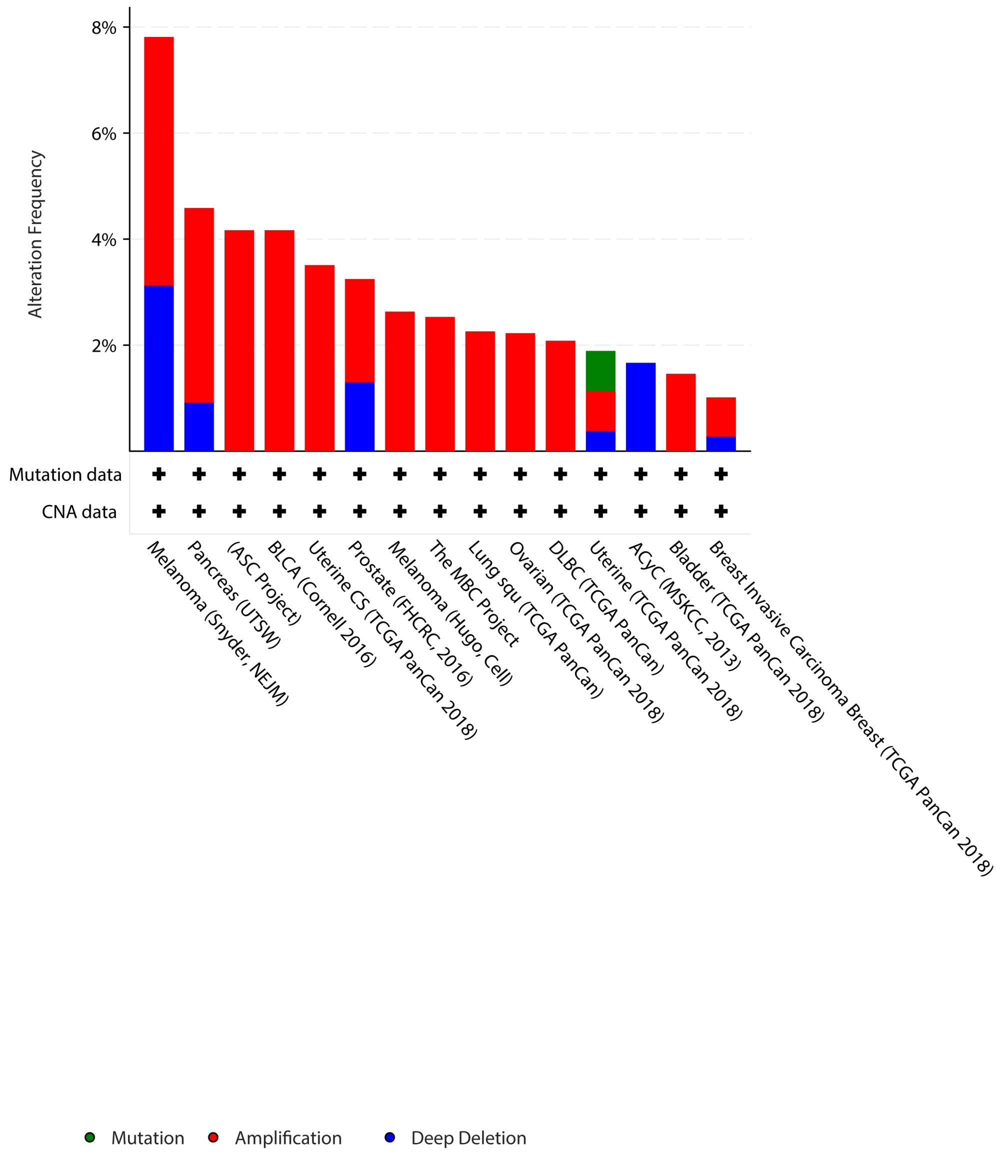

Supplementary Figure 10. *LINC01119 deregulations in cancer*. (**a**) Alteration frequency of LINC01119 in curated sets of non-redundant studies from TCGA (Alternation frequency >1%).

Unprocessed Blots Figure 4

Fig 4B

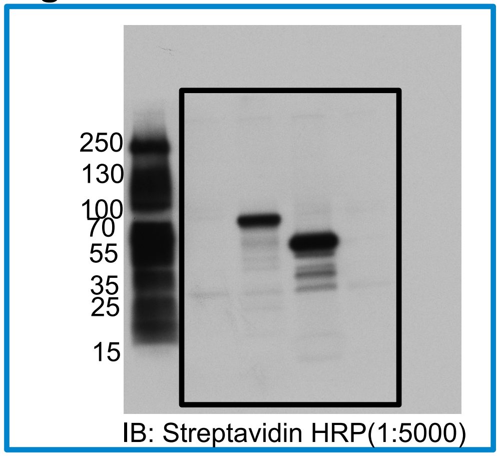

Fig 4C

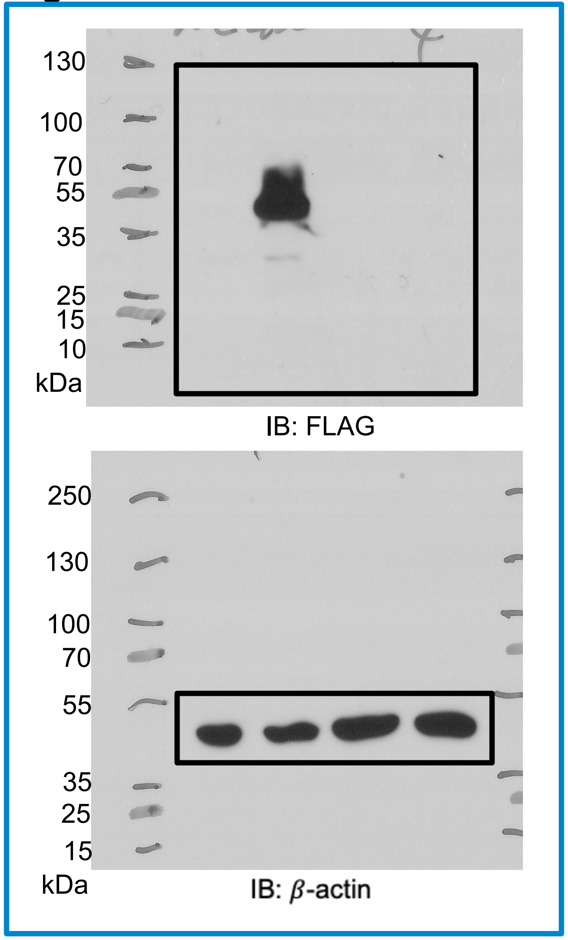

Fig 4D

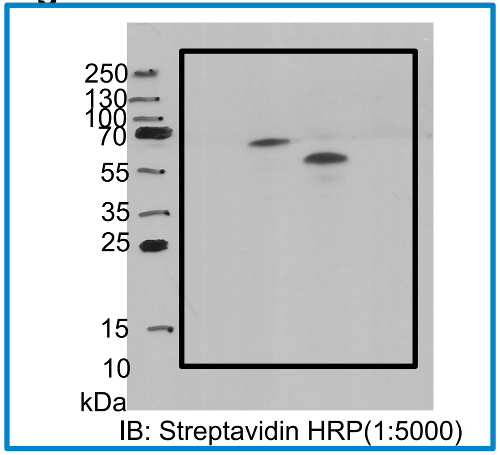

Unprocessed Blots Figure S8

Fig S8A

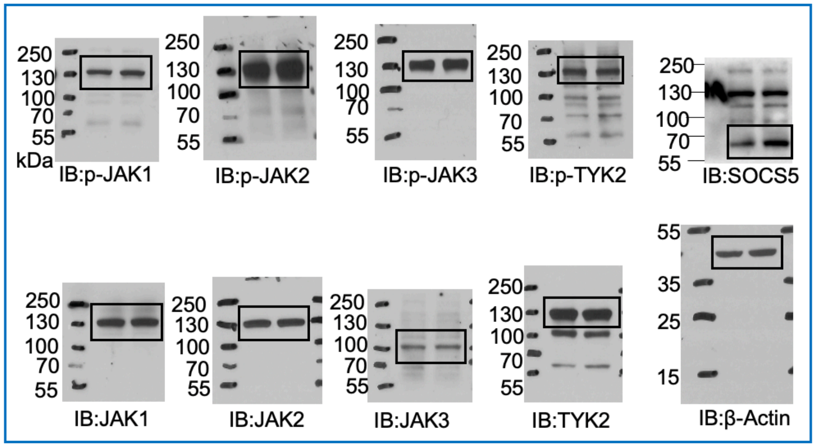

Fig S8B

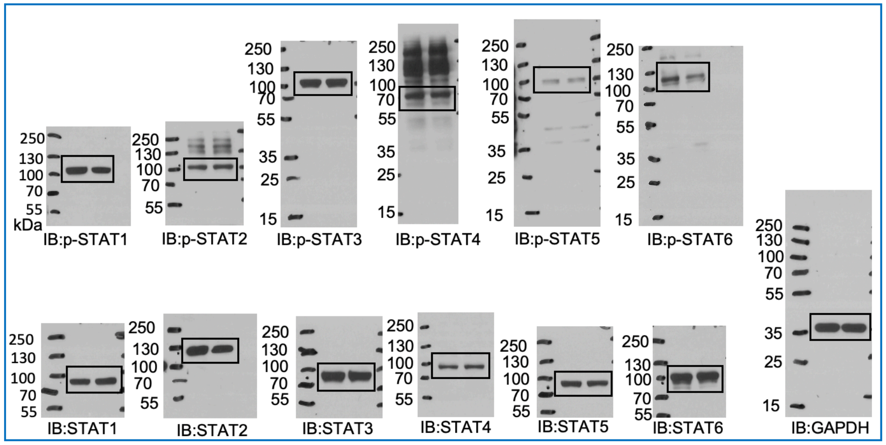

Fig S8C

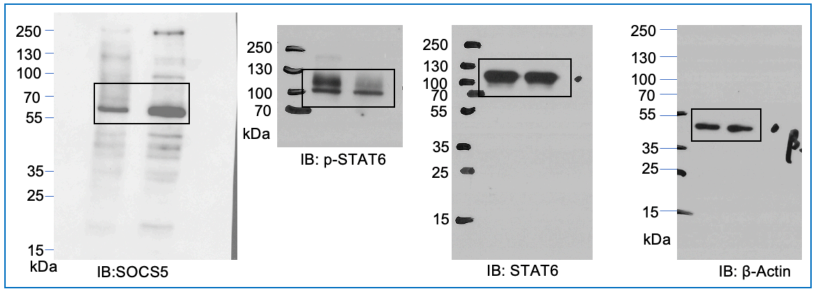

Fig S8D

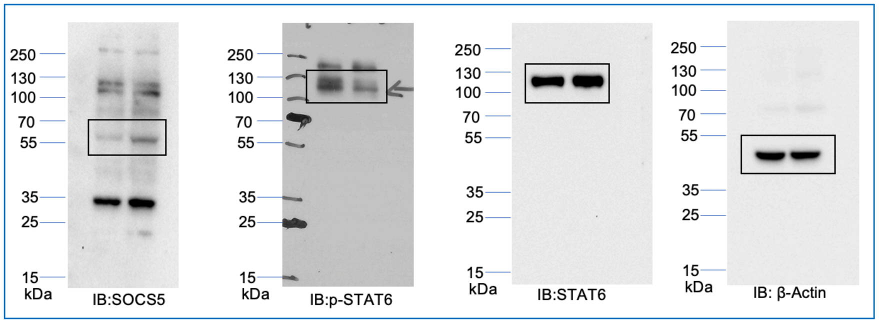

Fig S8E

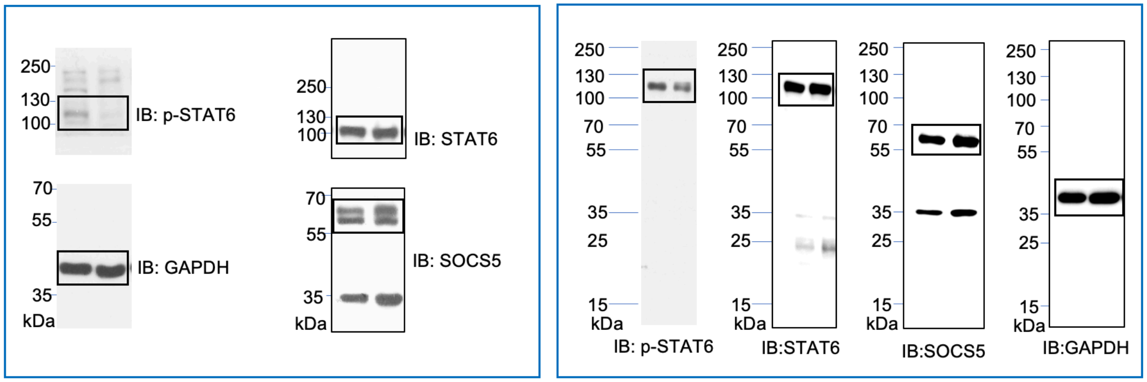

Unprocessed Blots Figure S9

Fig S9D

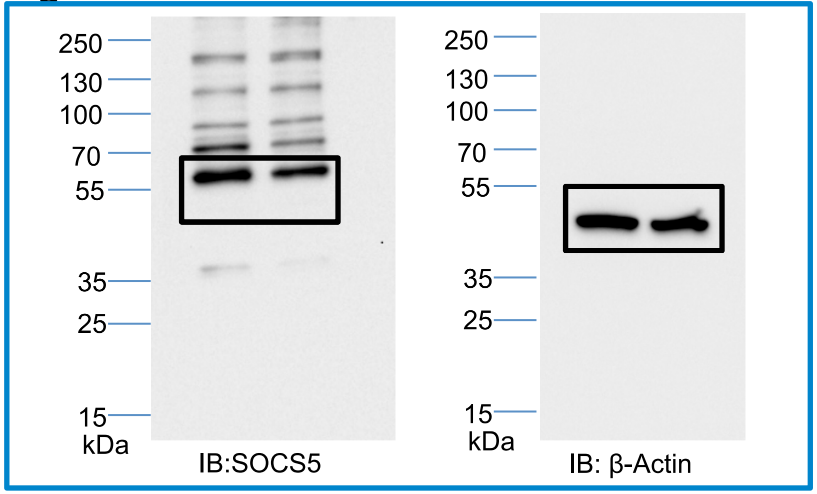

Fig S9E

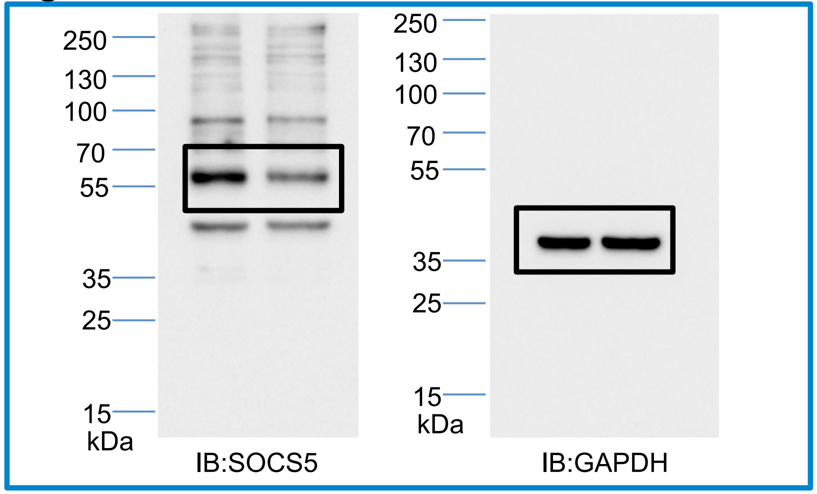

Supplement: Supplementary file 1 — Supplementary Information [file 41523_2021_259_MOESM1_ESM.pdf]
