# Supplementary material for: The LINC01119-SOCS5 axis as a critical theranostic in triple-negative breast cancer
Source: NPJ Breast Cancer. 2021 May 31;7:69. doi: 10.1038/s41523-021-00259-z (PMC8166834; doi:10.1038/s41523-021-00259-z)
Supplement: Supplementary file 2 — Reporting Summary [file 41523_2021_259_MOESM2_ESM.pdf]

nature research

Corresponding author(s): Antoine Karnoub

Last updated by author(s): Sep 23, 2020

Reporting Summary

Nature Research wishes to improve the reproducibility of the work that we publish. This form provides structure for consistency and transparency in reporting. For further information on Nature Research policies, see our Editorial Policies and the Editorial Policy Checklist.

Statistics

For all statistical analyses, confirm that the following items are present in the figure legend, table legend, main text, or Methods section.

n/a	Confirmed	
☐	☒	The exact sample size (*n*) for each experimental group/condition, given as a discrete number and unit of measurement
☐	☒	A statement on whether measurements were taken from distinct samples or whether the same sample was measured repeatedly
☐	☒	The statistical test(s) used AND whether they are one- or two-sided<br>*Only common tests should be described solely by name; describe more complex techniques in the Methods section.*
☐	☒	A description of all covariates tested
☐	☒	A description of any assumptions or corrections, such as tests of normality and adjustment for multiple comparisons
☐	☒	A full description of the statistical parameters including central tendency (e.g. means) or other basic estimates (e.g. regression coefficient) AND variation (e.g. standard deviation) or associated estimates of uncertainty (e.g. confidence intervals)
☐	☒	For null hypothesis testing, the test statistic (e.g. *F*, *t*, *r*) with confidence intervals, effect sizes, degrees of freedom and *P* value noted<br>*Give P values as exact values whenever suitable.*
☒	☐	For Bayesian analysis, information on the choice of priors and Markov chain Monte Carlo settings
☒	☐	For hierarchical and complex designs, identification of the appropriate level for tests and full reporting of outcomes
☒	☐	Estimates of effect sizes (e.g. Cohen's *d*, Pearson's *r*), indicating how they were calculated

Our web collection on statistics for biologists contains articles on many of the points above.

Software and code

Policy information about availability of computer code

Data collection	CFX384 Real-Time System from Bio-Rad was used to collect QRT-PCR data. ChemiDoc Touch Imaging System from Bio-Rad was used to collect immunoblotting data. Nikon ECLIPSE Ti-U system used to obtain pictures for RNAScope.
Data analysis	Graphpad Prism (version 8) and SPSS software (version 23) were used to do the graph figures and statistics. ImageJ 1.53 software was used to quantify colonies and Western blotting protein band intensity.

For manuscripts utilizing custom algorithms or software that are central to the research but not yet described in published literature, software must be made available to editors and reviewers. We strongly encourage code deposition in a community repository (e.g. GitHub). See the Nature Research guidelines for submitting code & software for further information.

Data

Policy information about availability of data

All manuscripts must include a data availability statement. This statement should provide the following information, where applicable:

- Accession codes, unique identifiers, or web links for publicly available datasets
- A list of figures that have associated raw data
- A description of any restrictions on data availability

LINC01119 expression was derived from TANRIC database (ibl.mdanderson.org) on TCGA data (LINC01119 was queried by position chr2:47055003-47086145). LINC01119 and SOCS5 expression levels in clinical breast cancer specimens were obtained from GENT2 (http://gent2.appex.kr/gent2/). LINC01119 (probe: 230799_at), SOCS5 (probe: 209648_x_at) and STAT6 (probe:201331_s_at) from CCLE (GSE36133), GSE28844, GSE16446, GSE102484, GSE12276, GSE76124 and GSE27830 derived from the GEO database (https://www.ncbi.nlm.nih.gov/geo/). Patient survival curves were conducted using the log-rank test and the proportional hazard model to compare KM survival curves (https://kmplot.com/). All data supporting the findings of this study are available from the corresponding author on reasonable request.

April 2020

Field-specific reporting

Please select the one below that is the best fit for your research. If you are not sure, read the appropriate sections before making your selection.

☒ Life sciences ☐ Behavioural & social sciences ☐ Ecological, evolutionary & environmental sciences

For a reference copy of the document with all sections, see nature.com/documents/nr-reporting-summary-flat.pdf

Life sciences study design

All studies must disclose on these points even when the disclosure is negative.

Sample size	Sample size was determined based on published papers and previous experience. For in vivo study, the samples are more than 5 per group. For in vitro studies, the samples are equal to or exceed 3 biological replicates.
Data exclusions	No data were excluded from our analyses.
Replication	Our experimental finds were confirmed with at least three independent replicates.
Randomization	All mice were randomly allocated into experimental groups. Standardized cell culture conditions were used to minimize variation.
Blinding	IHC analysis was performed in a double blinded manner by trained MD/PhDs. For others clinical assays, investigators were blinded to group allocation.

Reporting for specific materials, systems and methods

We require information from authors about some types of materials, experimental systems and methods used in many studies. Here, indicate whether each material, system or method listed is relevant to your study. If you are not sure if a list item applies to your research, read the appropriate section before selecting a response.

Materials & experimental systems

n/a	Involved in the study	
☐	☒	Antibodies
☐	☒	Eukaryotic cell lines
☒	☐	Palaeontology and archaeology
☐	☒	Animals and other organisms
☒	☐	Human research participants
☒	☐	Clinical data
☒	☐	Dual use research of concern

Methods

n/a	Involved in the study	
☒	☐	ChIP-seq
☒	☐	Flow cytometry
☒	☐	MRI-based neuroimaging

Antibodies

Antibodies used	The following antibodies were used for immunoblotting at 1:1000: FLAG (#14793), β-actin (#4970), GAPDH (#2118), JAK1 (#3344), p-JAK1(Y1034/1035) (#74129), JAK2 (#3230), p-JAK2 (Y1008) (#8082), JAK3 (#8827), p-JAK3 (Y980/981) (#5031), TYK2 (#14193), p-TYK2 (Y1054/1055) (#68790), STAT1 (#14994), p-STAT1 (Tyr 701) (#7649), STAT2(#72604), p-STAT2 (Tyr690) (#4441), STAT3 (#30835), p-STAT3 (Tyr705) (#9145), p-STAT3 (Ser727) (#9134), STAT4 (#2653), p-STAT4 (Tyr693) (#4134), STAT5 (#94205), p-STAT5 (Tyr694) (#4322), STAT6 (#5397), p-STAT6 (Tyr641) (#9361) from Cell Signaling Technology, and SOCS5 (WH0009655M1) and Vinculin (#V9131) from Sigma-Aldrich. Ki67 antibody (EPR3610; ab92742) was purchased from Abcam.
Validation	All the antibodies were purchased from Cell signaling Technology, Sigma-Aldrich, or Abcam, and these antibodies have been validated and detailed information could be found on the respective websites from manufacturers.

Eukaryotic cell lines

Policy information about cell lines

Cell line source(s)	MDA-MB-231, MDA-MB-468, HCC1937, BT20, HCC1143, BT549 and Hs578T cells were procured from American Type Culture Collection (ATCC). HCC70, T47D, ZR75, SUM149, CAL-51, and SUM159 were obtained from A. Toker (Beth Israel Deaconess Medical Center, Boston, MA), 4T1, 67NR, 4TO7, MCF7, and HEK293T cells from R. Weinberg (Whitehead Institute, Cambridge, MA), and MCF-10A from J. Brugge (Harvard Medical School, Boston, MA). Human primary breast cancer cells DT22 were a gift from D. El-Ashry (Sylvester Comprehensive Cancer Center, University of Miami Miller School of Medicine, Miami, FL). Bone marrow-derived human mesenchymal stem cells (BM-MSCs) were purchased from the Institute for Regenerative Medicine at Scott and White, Texas A&M Health Science Center (Temple, TX).

Authentication	All cell lines used in this study were authenticated by short tandem repeat DNA finger printing.
Mycoplasma contamination	Cell lines used in this study were routinely tested to be negative for mycoplasma
Commonly misidentified lines (See ICLAC register)	No commonly misidentified cell lines were used in this study.

Animals and other organisms

Policy information about studies involving animals; ARRIVE guidelines recommended for reporting animal research

Laboratory animals	Female 6-week old NCG mice were purchased from Charles River.
Wild animals	No wild animals were used in this study.
Field-collected samples	No field collected samples were used in this study.
Ethics oversight	All animal procedures were conducted under the approval of the IACUC at BIDMC.

Note that full information on the approval of the study protocol must also be provided in the manuscript.
